# Genomics of the Argentinian cholera epidemic elucidate the contrasting dynamics of epidemic and endemic *Vibrio cholerae*

**DOI:** 10.1038/s41467-020-18647-7

**Published:** 2020-10-01

**Authors:** Matthew J. Dorman, Daryl Domman, Tomás Poklepovich, Charlotte Tolley, Gisella Zolezzi, Leanne Kane, María Rosa Viñas, Marcela Panagópulo, Miriam Moroni, Norma Binsztein, María Inés Caffer, Simon Clare, Gordon Dougan, George P. C. Salmond, Julian Parkhill, Josefina Campos, Nicholas R. Thomson

**Affiliations:** 1 Wellcome Sanger Institute, Wellcome Genome Campus, Hinxton, CB10 1SA UK; 2 Department of Internal Medicine, Center for Global Health, University of New Mexico Health Sciences Center, Albuquerque, NM USA; 3 Instituto Nacional de Enfermedades Infecciosas, INEI-ANLIS “Dr. Carlos G. Malbrán”, Buenos Aires, Argentina; 4 London School of Hygiene and Tropical Medicine, London, WC1E 7HT UK; 5 Department of Medicine, Addenbrooke’s Hospital, University of Cambridge, Cambridge, CB2 0QW UK; 6 Department of Biochemistry, University of Cambridge, Cambridge, CB2 1QW UK; 7 Department of Veterinary Medicine, University of Cambridge, Cambridge, CB3 0ES UK

**Keywords:** Bacteriology, Bacterial genetics, Pathogens, Bacterial infection

## Abstract

In order to control and eradicate epidemic cholera, we need to understand how epidemics begin, how they spread, and how they decline and eventually end. This requires extensive sampling of epidemic disease over time, alongside the background of endemic disease that may exist concurrently with the epidemic. The unique circumstances surrounding the Argentinian cholera epidemic of 1992–1998 presented an opportunity to do this. Here, we use 490 Argentinian *V. cholerae* genome sequences to characterise the variation within, and between, epidemic and endemic *V. cholerae*. We show that, during the 1992–1998 cholera epidemic, the invariant epidemic clone co-existed alongside highly diverse members of the *Vibrio cholerae* species in Argentina, and we contrast the clonality of epidemic *V. cholerae* with the background diversity of local endemic bacteria. Our findings refine and add nuance to our genomic definitions of epidemic and endemic cholera, and are of direct relevance to controlling current and future cholera epidemics.

## Abstract (web summary)

Pandemic cholera was reintroduced to Argentina in 1992, leading to epidemic spread. Here, the authors use whole genome sequencing to show how, over 6 years, epidemic cholera was caused by invariant 7PET lineage *Vibrio cholerae*, against a background of sporadic disease caused by diverse local strains.

## Introduction

Latin America stands apart from the rest of the world in terms of its history of large-scale cholera epidemics^1–3^. There have been seven global cholera pandemics in recorded history, between 1819 and the present day^4^. Of these, the second pandemic (1829–1850) was the first to be seen in Latin America^5,6^. Between the 1830s and 1895, the region suffered from repeated cholera outbreaks, linked to global pandemics 2 through 5 (refs. ^4,6^). Importantly, although the sixth pandemic (1899–1923)^4^ affected most of Africa and Asia, Latin America did not experience cholera epidemics during this period, and thus was free of epidemic cholera for 96 years^6^. In 1991, during the seventh pandemic (1961–present), cholera returned to Latin America. Phylogeographic data have shown that this was attributable to the simultaneous introduction of two distinct sub-lineages of a globally-circulating phylogenetic lineage of *Vibrio cholerae* serogroup O1 biotype El Tor, dubbed 7PET^7–9^. An antimicrobial-sensitive sub-lineage of 7PET (LAT-1) was introduced into Peru in January 1991 and spread rapidly across South America^7,10,11^. Concurrently, a separate and distinct drug-resistant 7PET sub-lineage (LAT-2) was introduced into Mexico^1,7^. The Haitian cholera epidemic in 2010 was also caused by 7PET, albeit by a third, independently-introduced sub-lineage, LAT-3 (refs. ^7,8,12^).

**Fig. 1:**
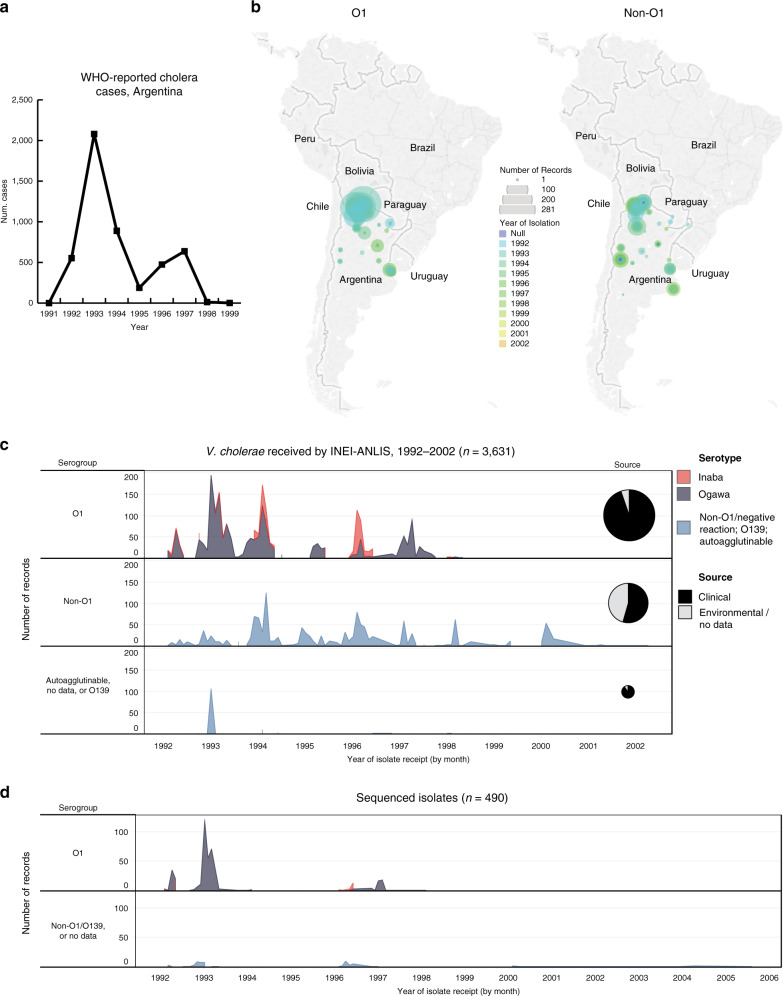
Geographic and temporal origin of *Vibrio cholerae* stored at INEI. Cholera cases were reported to WHO and PAHO throughout the epidemic period. **a** *V. cholerae* O1 and non-O1 were received by INEI from the North of Argentina. **b** The diameter of each circle scales with the number of isolates recorded to have originated from the indicated region. Circles are coloured by the year of receipt. Locations were inferred from the city (where recorded) or from the province which sent the isolate to INEI. These isolates were received between 1992 and 2002. **c** The *V. cholerae* O1 received were principally clinical isolates, whereas non-O1 *V. cholerae* were both clinical and environmental isolates. In our study, we sought to capture the genetic changes that occurred in *V. cholerae* O1 at the beginning and the end of the cholera epidemic (**d**), and included contemporaneous non-O1 isolates. Geographic data were unavailable for six O1 and 14 non-O1 *V. cholerae* (**b**); dates of isolation were not recorded for five *V. cholerae* (**c**). Four sequenced isolates had no date of isolation (**d**). It should be noted that the peaks in *V. cholerae* receipt in 1997–1998 predate Hurricane Mitch (October 1998), a natural disaster which led to the increase of cholera incidence in Central America^94^. Seven isolates from 2003 onwards that were sequenced as part of this study post-date the epidemic, and were not included in the metadata used to produce (**c**). Y-axes in (**c**) and (**d**): number of isolates received/sequenced. Publicly-available data were taken from^17–21^, https://www.who.int/cholera/statistics/en/, and http://ais.paho.org/phip/viz/ed_colera_casesamericas.asp, to produce (**a**).

Since Latin America had been free of cholera epidemics for 96 years, and because the major epidemics seen in the 1990s and in 2010 are attributed to the introduction of epidemic 7PET sub-lineages originating in South Asia^7^, Latin America presented a unique opportunity to understand the longitudinal evolution of pandemic *V. cholerae* upon its introduction into a naïve population. Argentina is an ideal setting in which to study the evolution of the pandemic clone during the 1990s because, unlike some other countries in the region, the socioeconomic position of Argentina^10,13^, and its preparedness for epidemics by the time cholera reached the country, are thought to have enabled the monitoring and control of the epidemic. Argentina instituted the mandatory notification of cholera cases nationwide during 1991 after cholera broke out in Peru^14^, and developed public information campaigns which resulted in a concomitant increase in the rate of diarrhoeal disease reporting^15^. This included changes to the Argentinian national diarrhoeal surveillance system, and the creation of the National Diarrhoea Network (formerly the National Cholera Network) which comprises 75 member laboratories as of 2014 (ref. ^16^). In addition, cumulative numbers of cholera cases and deaths in Argentina were reported to the World Health Organization (WHO) and the Pan American Health Organization (PAHO) (https://www.who.int/cholera/statistics/en/, http://ais.paho.org/phip/viz/ed_colera_casesamericas.asp^17–21^, Fig. 1a).

Following the beginning of the epidemic in Peru in January 1991, cholera spread to Argentina, with the first cholera cases reported in Salta province^18,22^, near to the border with Bolivia, on the 5th of February 1992 (refs. ^11,18^). Subsequently, Argentina reported cholera cases annually between 1992 and 1998 (refs. ^14,23–25^), with a total of 4,281 cases reported to WHO for this period^17–21^. Of these, an archive of over 3,500 phenotypically-characterised *V. cholerae* isolates is stored at INEI-ANLIS “Dr. Carlos G. Malbrán”, the national reference laboratory of Argentina. From previous genomic analyses looking across Latin America and including five Argentinian isolates^7,9^, it is thought that a single toxigenic *V. cholerae* clone belonging to the LAT-1 sub-lineage of 7PET was responsible for the Argentinian cholera epidemics, related to that which caused outbreaks in Peru^7^, and part of an epidemic which caused over 1.2 million cases of cholera across Latin America^13^. Therefore, we hypothesised that studying the Argentinian epidemic would elucidate the progression of an epidemic attributed to one discrete introduction of 7PET.

Given this unique set of circumstances, and that questions remain unanswered about how epidemic 7PET *V. cholerae* evolves over time after introduction to a region naïve to cholera, we sequenced nearly 500 *V. cholerae* isolates from the INEI collection. The collection is composed of all *V. cholerae* isolated from clinical suspected cholera cases as defined by the Ministry of Health between 1991 and 1998 (encompassing the epidemic period). During this time, all suspected cholera cases were tested in microbiology laboratories nationally, and putative *V. cholerae* were isolated and sent to INEI for further confirmation. After 1999, the National Diarrhoea and Foodborne Pathogens Network defined that one in every five stool samples should be tested for *Vibrio* spp., and all resultant *V. cholerae* isolates should be sent to INEI for further characterisation. The collection also includes *Vibrio* spp. from various environmental surveillance programmes, which had been collected to study the contribution of the environment to *Vibrio cholerae* dynamics in Argentina. Non-clinical environmental sources are defined by INEI as food, environmental and animal fodder; the majority of environmentally-isolated *V. cholerae* were derived from water sources, including drinking water.

We use these 490 clinical and environmental isolates and their associated records to link epidemiological reports of epidemic cholera in Argentina to genomic data. We also include non-O1 *V. cholerae* isolates of both clinical and environmental origins, to explore the underlying diversity of *V. cholerae* present in Argentina concurrent with the 1990s cholera pandemic, and to understand how these relate to those that caused the pandemic.

## Results

### Cholera outbreaks occurred annually in Argentina (1992–1997)

Between 1992 and 1998, WHO and PAHO data^17^ suggest that there were two peaks of cholera incidence in Argentina, one in 1993 (2,080 cases) and one in 1996/1997 (474 and 637 cases respectively) (Fig. 1a). However, there are discrepancies between these apparent maxima and other reports, which have suggested that there were seven epidemics of cholera in Argentina between 1992 and 1997 (refs. ^14,23–25^). To clarify this issue, we analysed the patterns of submission date for *V. cholerae* isolates sent to INEI. Between 10th February 1992 and 1st April 2002, INEI records show that the laboratory received at least 3,631 *V. cholerae* samples. Sixty-nine of these isolates were recorded as coming from countries other than Argentina: from Bolivia (*n* = 43), Chile (*n* = 16), Colombia (*n* = 1), Paraguay (*n* = 1), and Peru (*n* = 8). Four isolates have no recorded origin. The remaining 3,562 isolates originate from the North and Centre of Argentina (Fig. 1b), and may therefore represent over 82% of the PAHO/WHO-reported cholera cases for the whole country from this 1990s epidemic. Following the cholera outbreak in Peru, as part of epidemic preparedness, it was decided at the national level that every suspected cholera case reported in Argentina should be tested for *Vibrio cholerae* and the isolate sent to INEI from all microbiology laboratories from the network across the country, including the South region. *V. cholerae* was not reported by any of the laboratories from the South.

We used the metadata available for all *V. cholerae* samples received between 1992 and 2002, broken down by serogroup (O1/non-O1) and by serotype (Ogawa/Inaba). Of the isolates received, 2,189 were recorded as being of serogroup O1 (60.2%), and a total of 1,308 non-O1 *V. cholerae* were recorded. The vast majority (2,077, 94.8%) of *V. cholerae* O1 were of clinical origin, and 112 were either environmental isolates or their sources were not recorded. Of the non-O1 isolates, just 714 were of clinical origin (54.5%). From these data, it is clear that whilst there were periods during which no *V. cholerae* O1 were received (Fig. 1c), non-O1 *V. cholerae* were submitted to INEI more consistently during the 1990s, and their receipt rose coincidentally with peaks in *V. cholerae* O1 receipt. In addition, there were 134 isolates for which there were no serogroup data recorded (*n* = 129), that were autoagglutinable (*n* = 4), or were recorded as being of serogroup O139 (*n* = 1).

Figure 1c illustrates at least six peaks of *V. cholerae* O1 receipt within these data, occurring in early months of each year. This is consistent with previous reports, which allude to seven seasonal epidemics of cholera in Argentina during the 1990s^14,23–25^, and likely reflects the fact that WHO/PAHO data are only available as annual case/fatality numbers and are not broken down by month. *V. cholerae* serotype Ogawa dominated the number of received *V. cholerae* O1, with the exception of the incidence peak in early 1996. Ogawa isolates accounted for 1795 of all *V. cholerae* O1 (82.0%); 369 isolates were serotype Inaba (16.8%) (Fig. 1c). This is in agreement with previous reports, which indicated that *V. cholerae* serotype Ogawa was predominant in Argentina during the epidemic^26^, despite the initial cholera epidemic in Peru being ascribed to *V. cholerae* Inaba^17^. Twenty-five *V. cholerae* O1 (1.1%) did not have a serotype assigned (Fig. 1c). The peak of *V. cholerae* in January 1993 for which there were no serogroup data recorded (*n* = 106; 79% of all no-data isolates) coincides with a peak in *V. cholerae* O1 receipt (*n* = 196), and describes an outbreak of *V. cholerae* O1.

These data, particularly the shifts between Inaba and Ogawa serotype, suggested subtleties in the dynamics of cholera epidemics in Argentina during the 1990s that could not be understood from epidemiological data alone. We revived and sequenced the genomes of 490 archived *V. cholerae* isolates from INEI archives. These were chosen principally to capture diversity of both O1 and non-O1 *V. cholerae* at the beginning (1992–1993) and the end (1996–1997) of the Argentinian epidemic (Fig. 1d). The sequenced isolates were a spatiotemporally-broad cross-section of cholera incidence, from all regions of Argentina that experienced cholera cases, and were chosen to capture apparent shifts between Inaba and Ogawa serotype (Fig. 1c, d).

### The LAT-1 sub-lineage caused pandemic cholera in Argentina

**Fig. 2:**
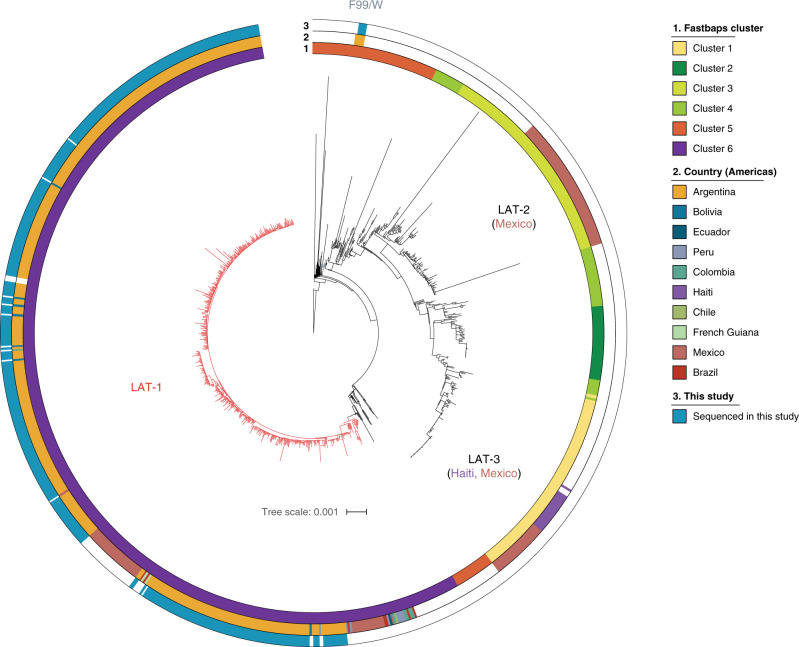
The vast majority of Argentinian 7PET *V. cholerae* O1 are members of the LAT-1 sub-lineage. A maximum-likelihood phylogeny produced for the 7PET lineage by calling SNVs between *V. cholerae* genome sequences and the N16961 reference genome sequence, rooted on M66, and excluding SNVs located in potentially recombined regions of the chromosome, for a total of 7,556 SNVs. Country of origin for genomes from the Americas are reported. All but four of the Argentinian 7PET genomes sequenced in this study are members of LAT-1 (in red). Clusters were determined using Fastbaps from an alignment of 3,874 parsimony-informative SNVs and used to delineate the LAT-1 sub-lineage (in red). Scale bar denotes the number of mutations *per* variable site.

Most of the sequenced *V. cholerae* isolates were found to be members of the 7PET phylogenetic lineage (425/490, 86.7%)^7^. These sequences were placed into phylogenetic context with 518 additional 7PET genomes^7^ (Supplementary Data 1; Fig. 2). The vast majority of sequenced Argentinian 7PET *V. cholerae* were members of LAT-1 (421/425, 99.05%), the sub-lineage introduced into Peru in 1991 (ref. ^7^) (Fig. 2). No Argentinian isolates were members of the LAT-2 sub-lineage, which was introduced into Mexico in the early 1990s. In addition to the LAT-1 isolates, four isolates which lacked the genes encoding the cholera toxin (i.e. were non-toxigenic) clustered together with F99/W, a previously-described non-toxigenic 7PET genome, also from Formosa^7,27^ (Fig. 2; Supplementary Data 1; Supplementary Figs. 1–3).

**Fig. 3:**
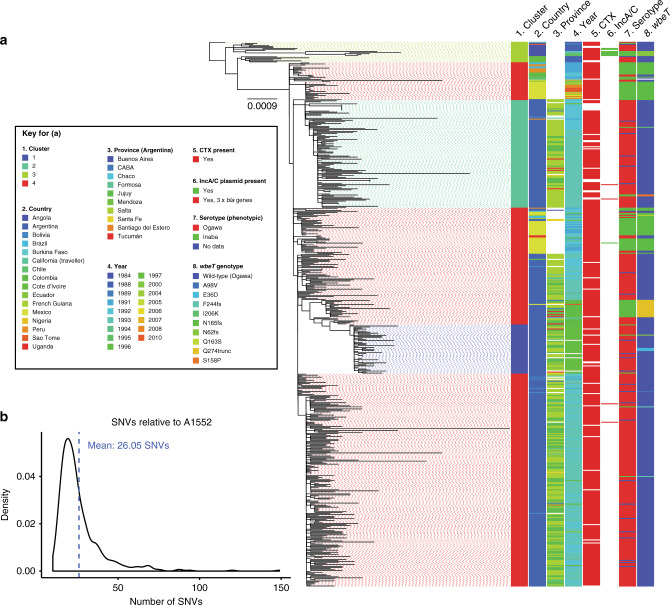
A phylogeny of the LAT-1 sub-lineage. **a** A maximum-likelihood phylogeny was calculated from 2,651 non-recombinant SNVs determined relative to the A1552 reference sequence (see Methods). The tree is rooted on the sequence of CNRVC980048, an isolate lacking WASA-1 from Burkina Faso, 1984. Scale bar denotes the number of mutations *per* variable site. Clusters were defined using Fastbaps using an alignment of 725 parsimony-informative SNVs. Metadata are reported where available; gaps are presented where data were unavailable, not determined, or recorded as no data. Initial figure produced using iCANDY and re-coloured manually (Adobe Illustrator CC). **b** Density plot illustrating the number of SNVs separating each of the LAT-1 isolates from the A1552 reference sequence. The blue line indicates the mean (26.05 SNVs). Interactive phylogeny available in Microreact (https://microreact.org/project/VAZD_K0kZ).

In order to obtain a LAT-1-specific phylogeny, we mapped the reads for the genomes in this sub-lineage, as well as the direct ancestors of LAT-1 and related genomes from Angola, Côte d’Ivoire, and Sao Tome^7,28^, to a closed genome assembly of strain A1552 (ref. ^29^). This Inaba *V. cholerae* O1 was isolated in 1992 from a Peruvian traveller^29,30^ and harbours the WASA-1 genomic island, a genetic hallmark of the LAT-1 sub-lineage^7,9^. An alignment of 2651 non-recombinant single nucleotide variants (SNVs) was used to calculate a maximum-likelihood phylogeny of these 532 genomes (Fig. 3a). We identified four genetic clusters using Fastbaps^31^, which were consistent with the topology of the phylogeny (Fig. 3a). Three clusters were specific to LAT-1 sequences from Latin America, and the fourth corresponded to the outgroup of sequences from Angola, Côte d’Ivoire, and Sao Tome (cluster 3, Fig. 3a).

Province-level geographic data were available for 475 of the 490 sequenced isolates, and for 416 of the 421 LAT-1 isolates (Fig. 3a; Supplementary Fig. 4). Sequences did not cluster by province or region. Strikingly, isolates from different Northern provinces were interspersed amongst one another, as were isolates from other countries in the region, such as Bolivia (Fig. 3a). However, we did observe limited clustering by date of isolation. Argentinian isolates from multiple provinces in 1996 and 1997 clustered phylogenetically, and isolates from 1997 occupied cluster 1 (Fig. 3a). Similarly, cluster 2 contains Argentinian genomes from 1992 to 1993 and one isolate from 1997, from multiple provinces, as well as one Bolivian and one Peruvian genome from 1992 to 1991 respectively (Fig. 3a; Supplementary Data 2, see Microreact project linked to this paper for an interactive map).

### The LAT-1 sub-lineage has limited genetic variation

Across all LAT-1 genomes and across the entire time period, we observed a striking lack of variation, at the levels of SNVs, gene gain/loss, and recombination. We found that just 0.03% of the A1552 genome was predicted to have undergone recombination (Supplementary Fig. 5), and a mean of only 26.05 non-recombinant SNVs across both chromosomes separated the sequence of each LAT-1 isolate from that of the A1552 reference genome (Fig. 3b) (min 10, max 149, stdev 14.10). Of the 2,651 SNVs in the LAT-1 alignment, 72.6% were private to single genomes in the data set. This result contrasted with genomic studies of 7PET in other settings; in hyper-endemic settings, six co-circulating sub-lineages of 7PET could be identified over a 4-year period^32^; in Africa, multiple 7PET sub-lineages have co-existed and replaced one another since 1970 (refs. ^28,33^), as has been observed in China^34,35^. This provided further evidence supporting the hypothesis that the Argentinian cholera epidemic was caused by one highly-clonal sub-lineage of 7PET.

We calculated a pangenome for LAT-1, and found that 3,368 genes were core to these isolates (present in ≥97% of isolates), suggesting that ~89% of the 3,776 annotated genes in the A1552 reference genome are core to LAT-1. Although gene gain and loss events were rare within LAT-1, there was evidence of the loss of the entire CTX bacteriophage within the data set, as it was absent from 51 of the isolates in the LAT-1 phylogeny (Fig. 3a, Supplementary Fig. 6A). It is possible that this loss was a result of long-term culture (some isolates were stored for 27 years before being sequenced) as has been noted previously^32,36^. We did not identify any candidate genes, which might have influenced the rapid spread of LAT-1. Rare and sporadic gene gains were also evident: four Argentinian LAT-1 *V. cholerae* carried extended-spectrum β-lactamase (ESBL) genes, *bla*_CTX-M-3_, *bla*_OXA-8_, and *bla*_TEM_ (Fig. 3a; Supplementary Figs. 7, 8). By manual inspection of the genome assemblies, we confirmed that these three ESBL genes were carried on contigs that also included IncA/C plasmid replicons (Fig. 3a; Supplementary Figs. 8, 9; Supplementary Data 2). Multidrug resistance plasmids have been seen in *V. cholerae* strains from Argentina previously^23,26^, Algeria^37^, and in Angolan isolates from 1988 and the early 1990s^7,28^, two of which did harbour *bla*_TEM_ (Supplementary Fig. 8; Supplementary Data 2). However, the complement of resistance determinants in these isolates does not match those found in Argentinian *V. cholerae* (Supplementary Fig. 8, Supplementary Data 2).

### Serotype variation and multiple *wbeT* mutations in LAT-1

The Inaba and Ogawa serotypes of *V. cholerae* O1 are commonly differentiated by diagnostic laboratories^38^ and by epidemiologists^39^ as well as in the formulation of cholera vaccines, such as Dukoral^40^, because both serotypes elicit different immunological responses^41^. Methylation of the terminal perosamine sugar on the O1 lipopolysaccharide chain by the WbeT enzyme confers an Ogawa phenotype; lack of methylation by disruption of *wbeT* confers an Inaba serotype^42–44^. There is evidence that reversion from Inaba to Ogawa serotype can occur in vivo, albeit rarely^43,45,46^.

In order to explain the apparent shifts between Inaba and Ogawa *V. cholerae* seen in Argentina (Fig. 1c), and more broadly in Latin America, we examined the sequence of *wbeT* in LAT-1. We identified nine distinct mutations in *wbeT* across LAT-1 which were predicted to disrupt the WbeT protein by non-synonymous, frameshift and truncating mutations (Fig. 3a; Supplementary Fig. 7; Supplementary Data 2, see Methods for full details). The genomic predictions correlated well with the longitudinal data detailed in Fig. 1c and with the phenotypic serotype assigned to each isolate; the *wbeT* genotype matched the phenotypic serotype for all but two of the 398 serotyped LAT-1 isolates sequenced in this study (99.4% concordance) (Fig. 3a; Supplementary Figs. 7, 8; Supplementary Data 2; Methods).

From previous reports, we know that the initial 1991 cholera epidemics in Peru and elsewhere in Latin America were associated with serotype Inaba *V. cholerae*, which became dominated by serotype Ogawa bacteria in 1992 and thereafter^47^. Genome sequences show that the Peruvian Inaba isolates from 1991 harbour the N165fs mutation in *wbeT* (Fig. 3a; Supplementary Fig. 7, Supplementary Data 2). Since LAT-1 was introduced into Latin America from West Africa^7^, we compared these data to West African Inaba isolates sharing a common ancestor with LAT-1, but collected between 1992 and 1995 in Angola, just after LAT-1 had been introduced into Peru^7,28^. These isolates were found to harbour a different mutation, N62fs (Fig. 3a; Supplementary Fig. 7). The N165fs mutation is present in 68 of the genomes of LAT-1 isolates collected since 1991. These include isolates from Brazil, Mexico, Chile, Argentina and Colombia, as well as isolates from Peru, all of which were originally serotyped as Inaba (Fig. 3a; Supplementary Figs. 7, 8; Supplementary Data 2). In addition, environmental isolates from Mexico, collected between 2004 and 2010, also harbour this mutation and are part of the same cluster of isolates (Fig. 3a; Supplementary Fig. 7). Hence, the N62fs and N165fs mutations are likely to have arisen independently, prior to spreading within West Africa and Latin America, respectively.

It has been hypothesised that cholera entered Argentina through the North of the country, which shares borders with Chile, Bolivia, Paraguay and Brazil^22^. Genomes from bacteria isolated in 1991 and 1992 from Chile, Bolivia and Brazil were included in our phylogeny^7,9^ (Fig. 3a). These were either Ogawa (Bolivia, *n* = 7; Brazil, *n* = 1) or Inaba (N165fs; Brazil, *n* = 6; Chile, *n* = 1) serotype. We found that these were interspersed amongst contemporaneous serotype Ogawa isolates, which were collected in Northern provinces of Argentina (Fig. 3a). All of these isolates were members of cluster 4, except for one Bolivian genome (1992) which was a member of cluster 2 (Fig. 3a). This observation, and the lack of genetic diversity within LAT-1, are consistent with the same *V. cholerae* sub-lineage circulating within, and between, countries at the Northern border of Argentina.

In 1996, cholera cases resurged in Argentina^17^ following a relative lull in 1995. This was associated with serotype Inaba *V. cholerae* (Fig. 1c). We found that 17 Argentinian Inaba *V. cholerae* isolates from 1996 formed a closely-related subclade within cluster 4 of the LAT-1 phylogeny (Fig. 3a), and harbour a unique mutation in *wbeT*, Q274trunc. This clade includes one 2010 Inaba isolate from Mexico (Fig. 3a; Supplementary Figs. 7 and 8). In addition, this subclade shares a common ancestor with the clade of isolates from 1997 which are serotype Ogawa and form a separate phylogenetic cluster (cluster 1, 48 isolates, Fig. 3a; Supplementary Figs. 7, 8). The 1996/1997 outbreak was not geographically-restricted; isolates from multiple provinces were part of this cluster (Fig. 3a).

### Non-7PET diversity contrasts with LAT-1 clonality

**Fig. 4:**
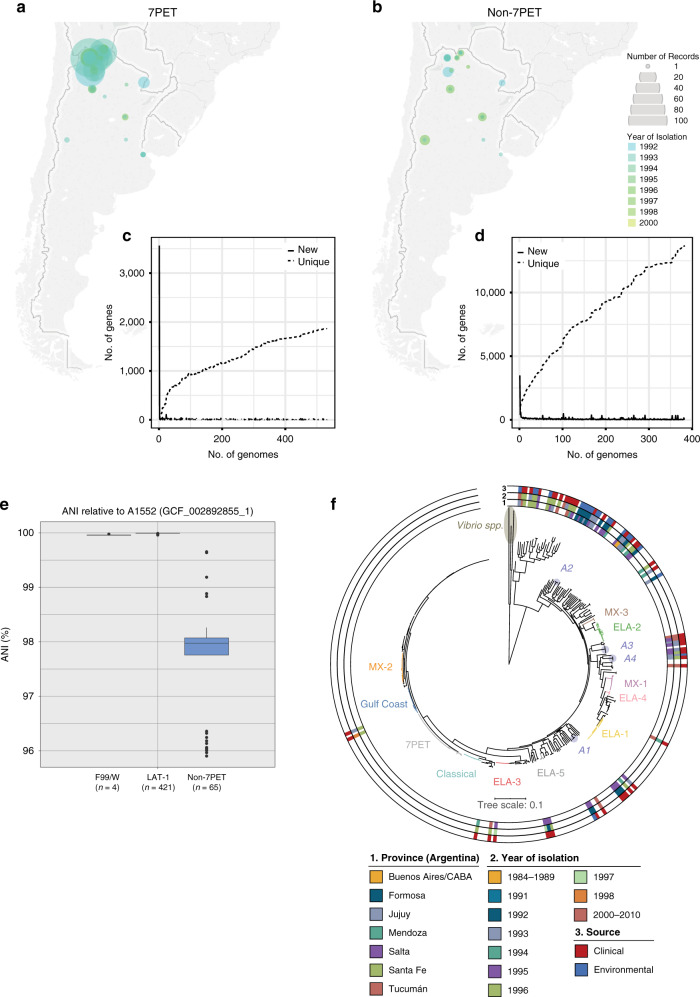
Contrasting the diversity of 7PET and non-7PET *V. cholerae* from Argentina. Maps depicting the year and place of origin of the isolates sequenced in this study are presented for 7PET and non-7PET isolates in (**a**) and (**b**), respectively. The non-7PET isolates were obtained from the same regions and years as the 7PET isolates. The number of new and unique genes added to the 7PET (LAT-1) pangenome and the non-7PET pangenomes are depicted in (**c**) and (**d**), respectively. The ANI for 7PET (both LAT-1 and F99/W) and non-7PET genomes sequenced in this study against the A1552 reference sequence are presented as a boxplot in (**e**). **f** Depicts a maximum-likelihood phylogeny calculated using 201,790 SNVs from a core-gene alignment of 2719 genes from 380 *V. cholerae* sequences, plus an outgroup of three related *Vibrio* species (383 sequences in total, Supplementary Data 3) on which the tree is rooted (brown). Lineages are named in line with previous reports^7^. Blue circles denote non-7PET lineages A1–A4. Scale bar denotes the number of mutations *per* variable site. **e** Boxplot details: Centre line = median, hinges = upper and lower quartiles, whiskers = 1.5 *interquartile range, outliers are plotted as datapoints.

Sixty-five isolates sequenced in this study were not members of 7PET, but were obtained from the same regions and times as the LAT-1 isolates (Figs. 1d, 4a, b; Supplementary Data 3). Thus, we placed these into context with a more diverse collection of *V. cholerae* sequences^7^, together with genomes from a recently-published study of non-epidemic *V. cholerae* O1 in China^48^, and then calculated a pangenome using these sequences (Fig. 4). The rate of gene discovery as sequences were added to the non-7PET pangenome was much greater than was observed in a LAT-1 pangenome, despite there being 38% more isolates in the LAT-1 pangenome (Fig. 4c, d). This indicates that genes are not being gained or lost by LAT-1. The non-7PET isolates were also extremely genetically diverse in comparison to the 7PET genomes, with a mean average nucleotide identity (ANI) relative to A1552 of 97.61 (min 95.90, max 99.65, stdev 0.960; Fig. 4e), in contrast to LAT-1 (mean ANI 99.99, min 99.96, max 99.998, stdev 0.0032; Fig. 4e). An ANI value of 95% is a common threshold for separating species^49^. The non-7PET isolates had a considerably expanded accessory genome when compared to LAT-1 (23,458 cloud genes in the collection of 383 diverse genomes compared to 3,313 in the 532 LAT-1 genomes) (Supplementary Fig. 6).

Of these 65 isolates, four were phenotypically and genotypically serogroup O1. Two were members of the previously-described Gulf Coast lineage of *V. cholerae* O1, including the single sequenced *V. cholerae* O1 from 1998. Both Gulf Coast isolates harboured CTX and were toxigenic, and the two remaining *V. cholerae* O1 isolates were members of ELA-3 (ref. ^7^) (Supplementary Fig. 10a; Supplementary Data 3). All four isolates were of clinical origin (Supplementary Data 3). The remaining 61 isolates lacked the genes required to produce cholera toxin, and were confirmed in silico not to harbour genes encoding the O1 antigen, though 45 of these were of clinical origin. We identified four new lineages of non-O1 non-7PET *V. cholerae* amongst these isolates, defined as clades formed by three or more Argentinian non-7PET isolates in the phylogeny, and denoted as A1–A4, where A stands for ‘Argentina’ (Fig. 4f). These lineages contained isolates that were of clinical origin alone (A1, A3) or clinical and environmental origin (A2, A4), were acquired in different years (A3, A4), and from different regions (A2, A3, A4), suggesting that these represent populations of non-7PET *V. cholerae* local to Argentina (Fig. 4f, Supplementary Data 3).

Of the 61 non-O1 non-7PET isolates, 21 harboured one of three distinct Type III secretion systems (Fig. 4f; Supplementary Data 3). These included the T3SS-2α described in *V. cholerae* AM_19226 (refs. ^50,51^), the less-common T3SS-2β system described by Carpenter et al.^51^, and a third putative T3SS system which most closely resembles genes present in the genomes of two virulent Chilean *Vibrio anguillarum* isolates^52^ (Supplementary Fig. 10). This putative T3SS was found in lineage A2. The presence of T3SS-2β in lineage A3 is of particular interest — this lineage is composed of clinical isolates, contains the previously-described Argentinian isolate TUC_T2734 (ref. ^7^), and includes one isolate from Salta province in the year 2000. T3SS elements were mutually exclusive and were never detected in the same genome at the same time (the distribution of T3SS is described in Supplementary Fig. 10 and Supplementary Data 3). From these limited data it is clear that more T3SS-positive non-7PET were of clinical origin than environmental (T3SS-2α: 10 clinical, 2 environmental; T3SS-2β: 5 clinical, 0 environmental; *V. anguillarum* element; 1 clinical, 3 environmental). It is also important to note that none of these T3SS are present in 7PET.

## Discussion

We contend that the observations described here could only have been made in Latin America and in Argentina. This is because the limited introductions of 7PET sub-lineages into the region, and the consequentially-enhanced surveillance schemes, provided a unique opportunity to understand the long-term evolutionary dynamics of epidemic *V. cholerae* evolving from point-source introductions on a continent where there have been millions of cholera cases from 1991 to present. This is the largest genomic study to date that investigates pandemic cholera in a single country, and we believe that it is the largest sequencing project designed to investigate the genomics of a single bacterial pathogen in Argentina. These data have had direct impact on national health policies in Argentina by changing the national alert system to distinguish between pandemic 7PET lineage and local *V. cholerae* using whole-genome sequencing. This recognises the increased risk presented by an outbreak of 7PET relative to that of a non-7PET outbreak, even if toxigenic or serogroup O1. This is to ensure that an epidemic response focuses on high-risk 7PET clones, and that there is also efficient monitoring of the contributions of endemic non-7PET clones to public health, including via environmental surveillance using a One Health approach.

Our data show that Ogawa *V. cholerae* from Argentina in 1992 are closely related to the Inaba isolates sequenced from Peru (Fig. 3a), and show that the Inaba/Ogawa shift observed in Peru and elsewhere in Latin America represented variation within LAT-1, rather than a separate introduction of another strain (Fig. 3a; Supplementary Fig. 8). Likewise, the outbreak of Inaba *V. cholerae* in Argentina in 1996 arose by mutation of *wbeT* from wild-type to an Inaba genotype (Q274trunc), and this mutation may have occurred in Argentina. The Q274trunc mutation is distinct from others identified within the data set, particularly the mutation associated with the Inaba phenotype in contemporaneous Mexican isolates (N165fs). This indicated that Argentinian cholera in 1996 was not caused by an introduction of a new Inaba (sub)lineage from elsewhere in Latin America; rather, LAT-1 *V. cholerae* that had already been introduced into Northern Argentina or the neighbouring countries acquired a new Inaba genotype and phenotype. The 1997 Argentinian outbreak, in turn, was caused by a close relative of the 1996 Inaba clone, but the topology of our phylogeny suggests that this was not a result of reversion from the Inaba Q274trunc genotype to an Ogawa genotype (Fig. 3a), adding nuance that would have been useful for public health at the time of the epidemic. These data underline that Ogawa/Inaba phenotypic variation is not phylogenetically informative, and may not be appropriate to use as an epidemiological marker.

Perhaps surprisingly, in spite of the sustained circulation of LAT-1, which was disseminated across Northern Argentina, an area of ~1.2 million km^2^ (Fig. 1b, Supplementary Fig. 4), these data suggest that very little genetic change, at the level of SNVs and gene gain/loss, occurred in this sub-lineage over a period of nearly 6 years. This lack of diversity is reminiscent of the clonality in other bacterial pathogens, such as *Salmonella* Typhi H58 (ref. ^53^). Our data also show that LAT-1 circulated amongst the countries at the Northern borders of Argentina during the early 1990s — for instance, cholera was first reported in Bolivia in August 1991 (refs. ^5,54^), and Bolivian genomes from the early 1990s are intermixed amongst the Argentinian genomes from 1992 (Fig. 3a; Microreact). The lack of clustering by region is likely to reflect the rapid dissemination of LAT-1 across South America.

LAT-1 invariance is juxtaposed with the diversity observed in non-O1 *V. cholerae* in Argentina (Figs. 3 and 4; Supplementary Fig. 6). Although *V. cholerae* research has tended to focus on studying epidemics and outbreaks, by definition, this tends to describe epidemic lineages. Non-7PET *V. cholerae* are highly variable, and as well as examples of local lineages of non-7PET *V. cholerae*, we also identified isolates which were confirmed microbiologically to be *Vibrio cholerae*, but were diverse phylogenetically and as measured by ANI values. Non-7PET *V. cholerae* continue to be associated with clinical cases of disease, but remain understudied. The disease associated with these isolates — and whether virulence determinants such as T3SS contribute to this disease — is the focus of future work, though there is evidence to suggest that T3SS contributes to diarrhoea caused by non-7PET *V. cholerae*^55^. We also see here, with the caveat of a small sample size, that the clinical non-7PET isolates were enriched for the presence of T3SS (16/21 isolates).

We show that a single clone of *V. cholerae* O1 (Fig. 2), now known to be one sub-lineage of 7PET^7^, was responsible for pandemic cholera in Argentina^2,3,25,56^, in spite of the seasonal fluctuations and serotype variation observed (Fig. 1c). Our genomic data provide fine-scaled insight into the evolution of LAT-1 that would not have been captured by routine surveillance alone. However, it is also important to state that these data validate fundamental observations made by public health authorities during the cholera epidemics of the 1990s — that the outbreaks in Argentina were caused principally by Ogawa *V. cholerae*, which were closely related to the Peruvian strain as determined by PFGE^1,25,47,57,58^. Furthermore, by including non-7PET *V. cholerae* in our study, we found a highly diverse population of this species in Argentina existing concurrently with the extremely invariant LAT-1 pandemic sub-lineage. We suggest that these non-7PET, which include O1 and non-O1 serogroup isolates, represent those *V. cholerae* that are truly endemic to Argentina, and are evolving locally but lack the propensity to cause epidemics and to spread in the same way as 7PET. Therefore, the fact that Latin America was cholera-free for 97 years was due solely to the absence of pandemic lineages, and it is a consequence of elevated surveillance due to the LAT-1 introduction that endemic *V. cholerae* were captured. In the absence of clinical data associated with these non-7PET isolates, we cannot determine whether they are aetiological agents of cholera, or of a cholera-like illness. However, it is clear that non-7PET *V. cholerae* were present, and associated with disease at a low level, in Argentina throughout the 1992–1998 cholera epidemic and thereafter (Fig. 1c).

It is vital to understand the diversity of the local, endemic *V. cholerae* that co-exist alongside 7PET during a cholera epidemic. This is because non-epidemic *V. cholerae* present in a country may contribute to cases of disease that are symptomatic of cholera, but do not pose the same relative risk to public health as 7PET. The case in the Chaco region during 2005, which was caused by non-epidemic *V. cholerae* O1 of the MX-2 lineage^7^ and failed to cause epidemic cholera, exemplifies this point. Similar observations have recently been made in China^48^. Relative risk of *V. cholerae* lineages should be accounted for in the magnitude of epidemic preparedness responses to such outbreaks, as is now being done in Argentina. The Global Task Force on Cholera Control has committed to reducing deaths from cholera by 90% before the year 2030 (ref. ^59^). This campaign focuses on the control of cholera, the disease, rather than on 7PET, the aetiological agent of epidemic cholera. As cholera control is implemented in countries that currently experience a high incidence of cholera attributed to 7PET, cases of cholera will decline. We anticipate that as epidemic cholera is reduced in magnitude, disease caused by non-7PET *V. cholerae* will become more visible, just as has been observed in Argentina. By using genomic definitions to differentiate pandemic lineages for public health epidemic preparedness responses, as is being done in Argentina, we propose that concerted control efforts including epidemiologists, public health authorities and microbiology laboratories targeting 7PET specifically, and accounting for background levels of endemic non-7PET disease, could see epidemic cholera eliminated in Latin America.

## Methods

### Bacterial strains and oligonucleotide primers

A list of bacterial isolates sequenced in this study is reported in Supplementary Data 1–3, together with relevant metadata and results from genomic analyses. Additional genome sequences used for contextualisation are listed in Supplementary Data 1–3. The sequences of oligonucleotides used for PCR (see Microbiology section below and Supplementary Methods) are detailed in Supplementary Table 1.

### Bacterial culture, genomic DNA isolation and sequencing

*Vibrio cholerae* isolates were revived from archived stocks on tryptic soy agar plates or in alkaline peptone water, and were subsequently passaged on thiosulfate-citrate-bile salts media to select for *V. cholerae*. Minimal biochemical tests (oxidase, Kligler tests) were performed to confirm *Vibrio* spp. identity. Genomic DNA extractions at INEI were carried out from 1 ml of liquid culture using the QiaCube Connect (Qiagen). Extractions carried out at the Sanger Institute were performed using the Epicentre Masterpure kit and a modified version of the manufacturer’s protocol^60 ^— briefly, single purified colonies of *V. cholerae* were used to lawn an LB agar plate. Loopfuls of bacterial lawns were resuspended in 300 μl Tissue & Cell Lysis Solution supplemented with Proteinase K, incubated at 65 °C for 20–25 min with intermittent vortexing to lyse cells, and then treated with RNase A for 30 min to remove contaminating RNA. Thereafter, the manufacturer’s protocol was followed to remove protein contaminants and to purify genomic DNA. Approximately 0.5 μg of genomic DNA *per* isolate was used for sequencing with the Illumina HiSeq X10 platform at the Wellcome Sanger Institute.

### Microbiology

Isolates were received and subjected to biochemical and molecular testing by INEI at the time of their receipt, principally during the 1992–1998 epidemic period. During the cholera epidemic, minimal biochemical tests (Kligler, oxidase, haemaaglutination and indole) and complementary tests (chloride and decarboxylase) were performed to verify the identity of *V. cholerae*. The production of cholera toxin was assessed using enzyme-linked immunosorbent (ELISA) assays^61^. Where required, PCR was performed to confirm an isolate as *V. cholerae*, as well as the presence of genes encoding *ctxA* and *tcpA*^62^, and other virulence factors. The oligonucleotide primer sequences and reaction conditions for these PCR tests are detailed in Supplementary Methods and Supplementary Tables 1–5. All of the microbiological and molecular assays used at INEI-ANLIS for the characterisation of *V. cholerae* are also described in a publicly-accessible protocol manual^63^.

### Sequence data quality control

A total of 21 sequenced isolates contained substantial amounts of contaminating sequences from non-*Vibrio* species, and were excluded from this study, for a total of 490 sequences used in this analysis. Contamination was assessed using Kraken^64^, by examining the overall length of the SPAdes assembly (data were summarised using assembly-stats v1.0.1 (https://github.com/sanger-pathogens/assembly-stats) and assemblies greater than 5 Mbp in length were excluded) and by inspection of initial phylogenetic trees.

### Genome assembly and annotation

Illumina sequencing reads were assembled using SPAdes v3.8.2 (ref. ^65^) as part of a high-throughput analysis pipeline^66^, and annotated using Prokka v1.5 (ref. ^67^). External publicly-available sequences were similarly assembled from raw sequencing reads — where these were not available, assemblies were downloaded from Genbank and annotated using Prokka v1.5 for uniformity within the data set.

### Pangenome analysis

Pangenomes were calculated using annotated genome sequences for a diverse collection of *V. cholerae* (Supplementary Data 3) as well as the LAT-1 subset of a 7PET-specific data set (Supplementary Data 2). Roary v3.12.0 (ref. ^68^) was used for these calculations, with options ‘-e–mafft -s -cd 97′. For the non-7PET genome collection, an alignment of 2719 core genes was used for phylogenetic analysis (see below).

### SNV identification and phylogenetic analysis

For 7PET and LAT-1 phylogenetic analyses, sequencing reads were mapped to reference genomes (accession numbers LT907989/LT907990 for N16961; CP025936/CP025937 for A1552) using SMALT v0.7.4 (http://www.sanger.ac.uk/science/tools/smalt-0). The reference was indexed using SMALT using a kmer size of 20 and a step size of 13 (-k 20 -s 13), and the reads were aligned using default parameters but with the maximum insert size (i) set as three times the mean fragment size of the sequencing library (target insert size 450 bp). PCR duplicate reads were identified using Picard v1.92 (https://broadinstitute.github.io/picard/) and flagged as duplicates in the BAM file. High-quality single nucleotide polymorphisms, including small indels, were identified as described by Harris et al.^69^ and reported previously for *V. cholerae*^7,28^. Briefly, BCF files of all variant sites were generated using samtools mpileup v0.1.19 (ref. ^70^) (parameters -d 1000 -DSugBf) and bcftools v0.1.19 (http://samtools.github.io/bcftools/). The bcftools option to call genotypes at variant sites was used. The following bcftools cut-off thresholds were applied: quality <50, map_quality <30, af1 < 0.95, ratio <0.75, depth <4, depth_strand <2, strand_bias <0.001, map_bias <0.001 and tail_bias <0.001. If any of these filters were not met, the base was called as uncertain. A pseudo-genome was constructed by substituting the base call at each site (variant and non-variant) in the BCF file into the reference genome. Uncertain sites were substituted with an N. Insertions with respect to the reference genome were ignored and deletions with respect to the reference genome were filled with N’s in the pseudo-genome to keep it aligned and the same length as the reference genome.

Regions of the genome which were predicted to be recombined, and which might therefore affect the topology of calculated phylogenies, were identified and removed from the pseudogenome alignment using Gubbins v1.4.10 (ref. ^71^). Alignments consisting entirely of variable nucleotides were produced from whole-genome alignments using SNP-sites v2.4.1 (ref. ^72^). The non-7PET core-gene alignment was trimmed using trimAl v1.4.1 (ref. ^73^), and SNP-sites v2.5.1 was used to produce an alignment of 201,790 variable nucleotides. Maximum-likelihood phylogenetic trees were then calculated from SNV-only alignments using IQ-Tree v1.6.10 (ref. ^74^) under the general time reversible (GTR) and ascertainment bias correction (ASC) models^75^. Five thousand approximate likelihood ratio tests^76^ and ultrafast bootstrap approximations^77^ were performed to assess the robustness of the computed phylogenies.

LAT-1 genomes were clustered using Bayesian hierarchical clustering and partitioned using the Dirichlet Process Mixture model with Fastbaps v1.0.1 (ref. ^31^), run using default parameters. Fastbaps was similarly used to cluster sequences in the *V. cholerae* species phylogeny, using the Bayesian Hierarchical Clustering prior^78^ and excluding the three outgroup sequences from the alignment. Parsimony-informative SNVs were extracted from SNV-only alignments using extract_PI_SNPs.py (https://gist.github.com/jasonsahl/9306cd014b63cae12154) and these alignments were used as the input for Fastbaps. SNV distance matrices were calculated from SNV-only alignments using snp-dists v0.4 (https://github.com/tseemann/snp-dists). Average nucleotide identity (ANI) values were calculated using FastANI v1.0 (ref. ^49^).

### Detection of plasmid replicons, antimicrobial resistance genes, *ctxB* variants and in silico serotype assignment

*wbeT* and *ctxB* genotypes were assigned using ARIBA v2.12.1 (ref. ^79^) and a custom database consisting of the *ctxB* nucleotide sequence from N16961 (LT907989/LT907990) and the intact *wbeT* sequence from NCTC 9420 (ref. ^80^) (CP013319/CP013320), which translates into a protein sequence which is 100% identical to the WbeT sequence from the Ogawa isolate VX44945 (AEN80191.1)^81^. We assumed that an Inaba phenotype would be conferred on isolates in which ARIBA^79^ was unable to detect or assemble *wbeT* in its totality, and if a mutation in *wbeT* was detected that was predicted to frameshift or truncate translated *wbeT* (N62fs, N165fs, F244fs, Q274trunc), was associated with Inaba phenotypes (I206K), or was otherwise known to confer an Inaba phenotype (S158P^82^). We assumed, since none of the isolates harbouring the E36D *wbeT* mutation had an Inaba phenotype, that this mutation does not result in abolition of an Ogawa serotype. The sequences of other virulence genes were taken from the sequence of N16961 (LT907989/LT907990) or of the Classical *V. cholerae* isolate O395 (CP000626/CP000627). We confirmed using BLASTn and the pangenome gene presence/absence matrix that the WASA-1 genomic island, a marker characteristic of the LAT-1 sub-lineage^9,32^, was present in the assemblies for these sequences. Plasmid replicons and antimicrobial resistance genes were detected using ARIBA, the ResFinder database^83^, and the PlasmidFinder database^84^ (both databases accessed on 23/06/2019). The presence and absence of the *V. cholerae* serogroup O1 biosynthesis operon in non-7PET genomes was confirmed using the pangenome gene presence/absence matrix, and by testing for the presence of the O1 biosynthesis operon sequence using BLASTn (co-ordinates 234,000–286,000 in the N16961 reference genome, accession LT907989).

### Data visualisation

Data were visualised and maps were annotated using Tableau Desktop 2018.31. Maps in Tableau were produced using OpenStreetMap (^©^ OpenStreetMap contributors) which is licenced under a CC-BY-SA licence (https://www.openstreetmap.org/copyright). Phylogenetic trees were visualised using Figtree v1.4.3 (http://tree.bio.ed.ac.uk/software/figtree/) and iTOL v3 (ref. ^85^). Gene presence/absence matrices were visualised using roary_plots.py v0.1.0 (https://github.com/sanger-pathogens/Roary/tree/master/contrib/roary_plots). Other figures were produced using R v3.5.1 with the ggplot2 v3.1.1 (ref. ^86^) and reshape v0.8.8 (ref. ^87^) packages, Artemis v16 (ref. ^88^), ACT v13 (ref. ^89^), DNAplotter v1.11 (ref. ^90^), the Phandango web server^91^, Easyfig v2.2.2 (ref. ^92^), and iCANDY (https://github.com/simonrharris/iCANDY). Where figures were edited manually, this was performed using Adobe Illustrator CC v23.0.4.

### Ethics

Not applicable. This study uses archived bacterial samples processed by INEI. No identifiable data were available or used in this study.

### Reporting summary

Further information on research design is available in the Nature Research Reporting Summary linked to this article.

## Supplementary information

Supplementary material

Supplementary Data 1

Supplementary Data 2

Supplementary Data 3

Reporting summary

## Data Availability

All next-generation sequencing data generated in this study have been deposited into the European Nucleotide Archive (ENA; http://www.ebi.ac.uk/ena) under accession number ERP118963 [https://www.ebi.ac.uk/ena/browser/view/PRJEB35844]. The assemblies which were used to produce Supplementary Figs. 9, 10B have been deposited into the ENA as part of ERP118963 [https://www.ebi.ac.uk/ena/browser/view/PRJEB35844]. The original data which underpin Fig. 1 are held and maintained by INEI-ANLIS, a National Reference Laboratory of Argentina. Therefore, in order to maintain confidentiality and protect privacy, these raw data have not been published with the article, but managed access to these records can be facilitated on request (via Dr. J.C., senior author). All other data used to generate figures in this paper, including sequence alignments, phylogenetic trees, and data matrices, are available in Figshare [10.6084/m9.figshare.11310131] or in the Supplementary Data associated with this paper. An interactive LAT-1 phylogeny is available in Microreact^93^ (https://microreact.org/project/VAZD_K0kZ).

## Code availability

The R code used to produce the initial versions of Figs. 3b, and 4e is available in Figshare [10.6084/m9.figshare.11310131].
