# Supplementary material for: Genomics of the Argentinian cholera epidemic elucidate the contrasting dynamics of epidemic and endemic Vibrio cholerae
Source: Nat Commun. 2020 Oct 1;11:4918. doi: 10.1038/s41467-020-18647-7 (PMC7530988; doi:10.1038/s41467-020-18647-7)
Supplement: Supplementary file 1 — Supplementary material [file 41467_2020_18647_MOESM1_ESM.pdf]

Supplementary material for

Genomics of the Argentinian cholera epidemic elucidate the contrasting dynamics of epidemic and endemic *Vibrio cholerae*

Dorman *et al.*

This file includes:

Supplementary Methods

Supplementary Tables 1-5

Supplementary Figures 1-10

Supplementary References

Supplementary Methods

PCR conditions

PCR was performed using primers listed in Supplementary Table 1. Multiplex reactions to identify an isolate as *V. cholerae* and determine the presence and absence of *ctxA* and *tcpA* were carried out according to the scheme in Supplementary Table 2. Separate multiplex PCR reactions were carried out to classify an isolate as serogroup O1 or O139 (Supplementary Table 3), and to determine the presence or absence of virulence genes *stn/o* and *rtxA* (Supplementary Table 4). The presence or absence of *tcpI* was also determined by PCR (Supplementary Table 5).

These reactions are also detailed in a publicly-accessible INEI-ANLIS procedural manual [1].

Supplementary Table 1. Oligonucleotide primer sequences used to characterise *V. cholerae* isolates by PCR.

**Primer ID**	**Sequence (5'-3')**	**Primer target; Reference**
VCO1-F2	CAACAGAATAGACTCAAGAA	O1/O139, multiplex reaction ; [2]
VCO1-R2	TATCTTCTGATACTTTTCTAC	
VCO139-F2	TTACCAGTCTACATTGCC	
VCO139-R2	CGTTTCGGTAGTTTTTCTGG	
VC-F2	TTAAGCSTTTTCRCTGAGAATG	*V. cholerae* 16S-23S rRNA ; [3]
VCm-R1	AGTCACTTAACCATACAACCCG	
CT 94-F	CGCGCAGATTCTAGACCTCCTG	*ctxA* ; [2]
CT 614-R	CGATGATCTTGGAGCATTCCCAC	
TCP 72-F	CACGATAAGAAAACCGGTCAAGAG	*tcpA*$^{El\ Tor}$ [2]
TCP 477-R	CGAAAGCACCTTCTTTCACGTTG	
stn/o 67-F	TCGCATTTAGCCAAACAGTAGAAA	*stn/o* ; [4]
stn/o 194-R	GCTGGATTGCAACATATTTCGC	
rtxA-F	CTGAATATGAGTGGGTGACTTACG	*rtxA* ; [5]
rtxA-R	GTGTATTGTTCGATATCCGCTACG	
tcpI 132-F	TAGCCTTAGTTCTCAGCAGGCA	*tcpI* ; [4]
tcpI 951-R	GGCAATAGTGTCGAGCTCGTTA	

Supplementary Table 2. Multiplex PCR scheme for *V. cholerae* / *ctxA* / *tcpA* detection.

**Reaction composition**		
**Reagent**	**Volume (µl)**	**Concentration**
H_2O	9.3	-
Tris-HCl buffer 10 X	2.5	1 X
$MgCl_2$ (50 mM stock)	1	2 mM
dNTP (mix; 2.5 mM stock)	2	0.2 mM
primer VC-F2	2	0.8 µM
primer VC-mR1	2	0.8 µM
primer CT 94-F	1	0.4 µM
primer CT 614-R	1	0.4 µM
primer TCP 72-F	1	0.4 µM
primer TCP 477-R	1	0.4 µM
Taq DNA polymerase 5 U/µl	0.2	1 U
DNA	2	-
Final volume (µl)	25	-
**Reaction conditions**		
**Step**	**Temperature (˚C)**	**Time**
Denature	94	2 min
30 cycles	94	45 s
	60	45 s
	72	45 s
Final extension	72	10 min
Hold	14	

Supplementary Table 3. Multiplex PCR scheme for O1/O139 serogroup detection.

**Reaction composition**		
**Reagent**	**Volume (μl)**	**Concentration**
H_2O	13.6	-
Tris-HCl buffer 10 X	2.5	1 X
$MgCl_2$ (50 mM stock)	0.75	1.5 mM
dNTP (mix; 2.5 mM stock)	2	0.2 mM
primer VCO1-F2	1	0.4 μM
primer VCO1-R2	1	0.4 μM
primer VCO139-F2	1	0.4 μM
primer VCO139-R2	1	0.4 μM
Taq DNA polymerase 5 U/μl	0.15	0.75 U
DNA	2	-
Final volume (μl)	25	-
**Reaction conditions**		
**Step**	**Temperature (°C)**	**Time**
Denature	94	2 min
30 cycles	94	1 min
	55	1 min
	72	2 min
Final extension	72	10 min

Supplementary Table 4. Multiplex PCR scheme for *stn/o* and *rtxA* detection.

**Reaction composition**		
**Reagent**	**Volume (µl)**	**Concentration**
H_2O	8.55	-
Tris-HCl buffer 10 X	2.5	1 X
$MgCl_2$ (50 mM stock)	0.75	1.5 mM
dNTP (mix; 2.5 mM stock)	2	0.2 mM
primer *stn/o* 67-F	2.5	1 µM
primer *stn/o* 194-R	2.5	1 µM
primer *rtxA*-F	2	0.8 µM
primer *rtxA*-R	2	0.8 µM
Taq DNA polymerase 5 U/µl	0.2	1 U
DNA	2	-
Final volume (µl)	25	-
**Reaction conditions**		
**Step**	**Temperature (˚C)**	**Time**
Denature	94	2 min
30 cycles	94	45 s
	55	45 s
	72	45 s
Final extension	72	10 min

Supplementary Table 5. PCR scheme for *tcpI* detection.

**Reaction composition**		
**Reagent**	**Volume (µl)**	**Concentration**
H_2O	13.63	-
Tris-HCl buffer 10 X	2.5	1 X
$MgCl_2$ (50 mM stock)	0.75	1.5 mM
dNTP (mix; 2.5 mM stock)	2	0.2 mM
primer *tcpI* 132-F	2	1 µM
primer *tcpI* 951-R	2	1 µM
Taq DNA polymerase 5 U/µl	0.125	1 U
DNA	2	-
Final volume (µl)	25	-
**Reaction conditions**		
**Step**	**Temperature (˚C)**	**Time**
Denature	94	2 min
30 cycles	94	1 min
	60	1 min
	72	2 min
Final extension	72	10 min

Supplementary Figures

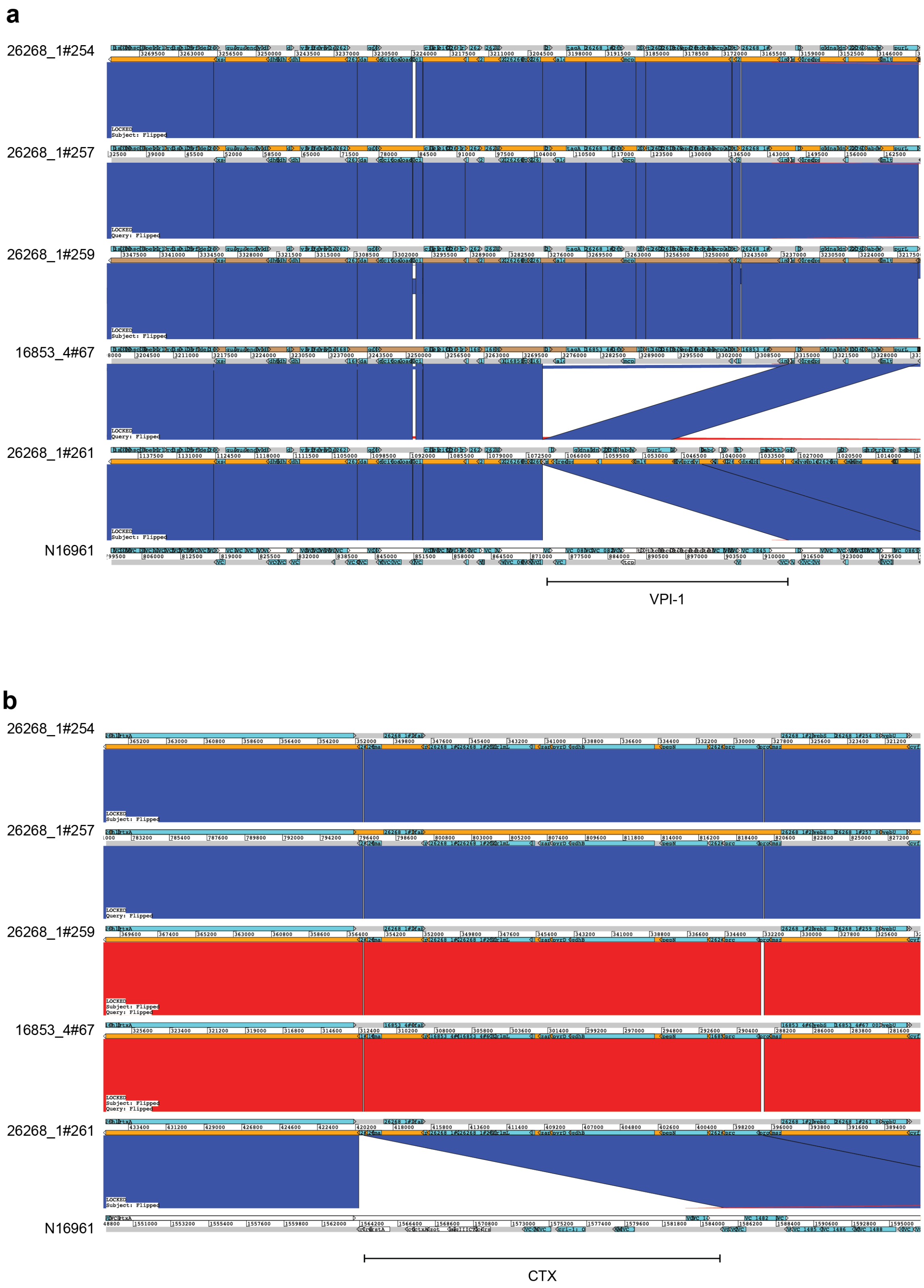

Supplementary Figure 1. ACT [6] (BLASTn) was used to compare synteny between the N16961 reference sequence and the assemblies for the five F99/W genomes at the VPI-1 and

CTXφ integration loci. Each of the F99/W isolates lacks CTXφ, and all but one harbour VPI-1. The absence of VPI-1 from sequence CCBT0194 was also validated by mapping (Supplementary Figure 3).

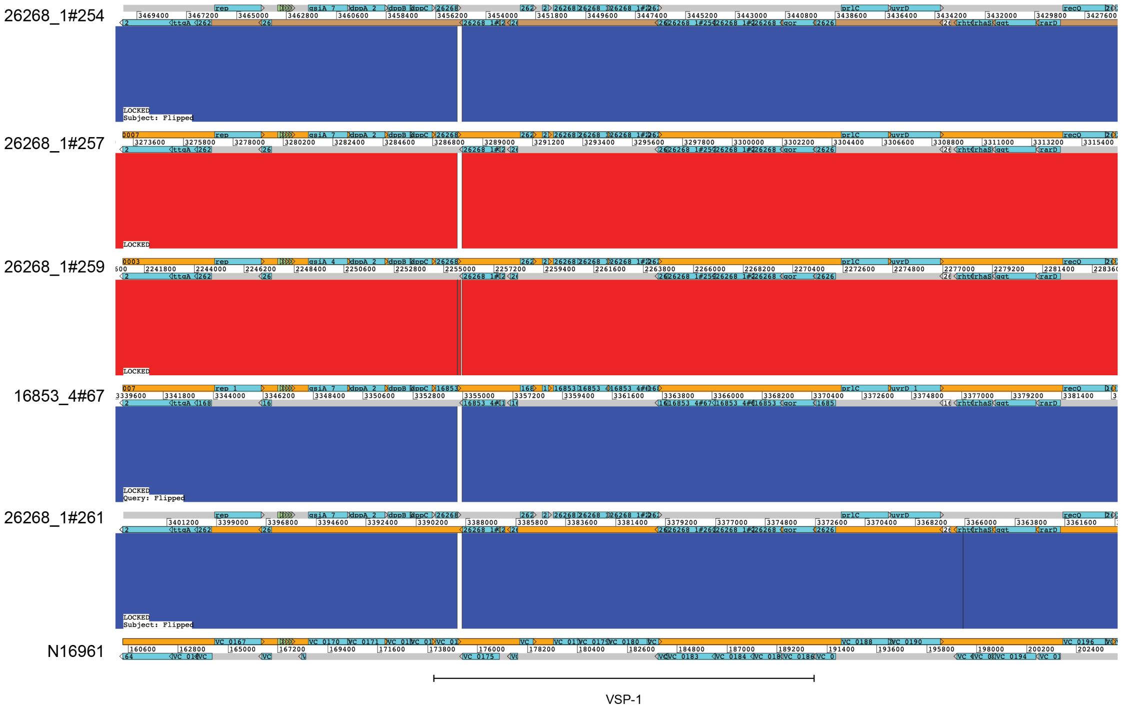

Supplementary Figure 2. ACT comparisons of synteny [6] (BLASTn) between the N16961 reference sequence and the assemblies for the five F99/W genomes illustrate that members of the F99/W clade possess VSP-1, and in the same chromosomal location as in N16961.

a

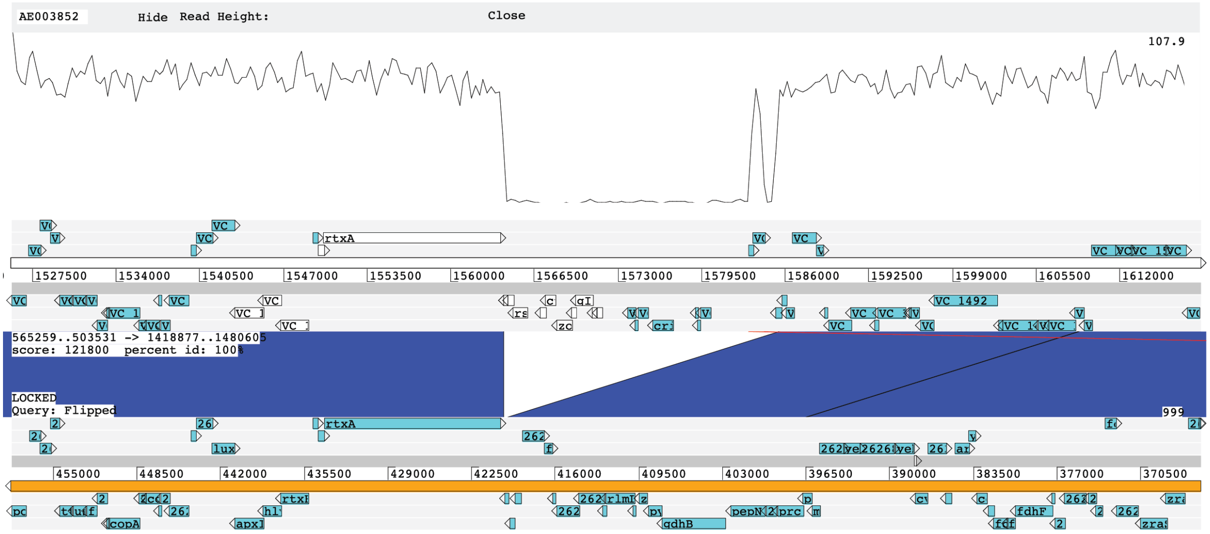

b

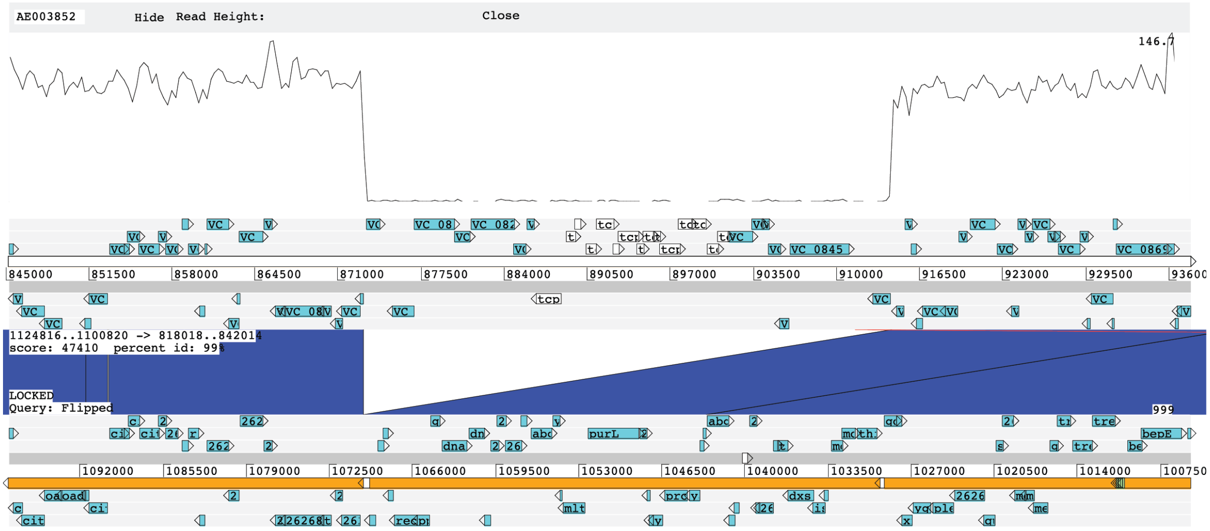

Supplementary Figure 3. Confirmation that CTXφ (A) and VPI-1 (B) are both absent from 26268_1#261. Presented are ACT comparisons (BLASTn) between the N16961 reference sequence and the assembly for sequence CCBT0194, as well as mapping plots in which the reads from CCBT0194 were mapped to N16961. Scale is as indicated. These genomic islands are absent from this genome assembly, and the assembly is not broken at these sites. Figure produced using Artemis and BamView [7,8].

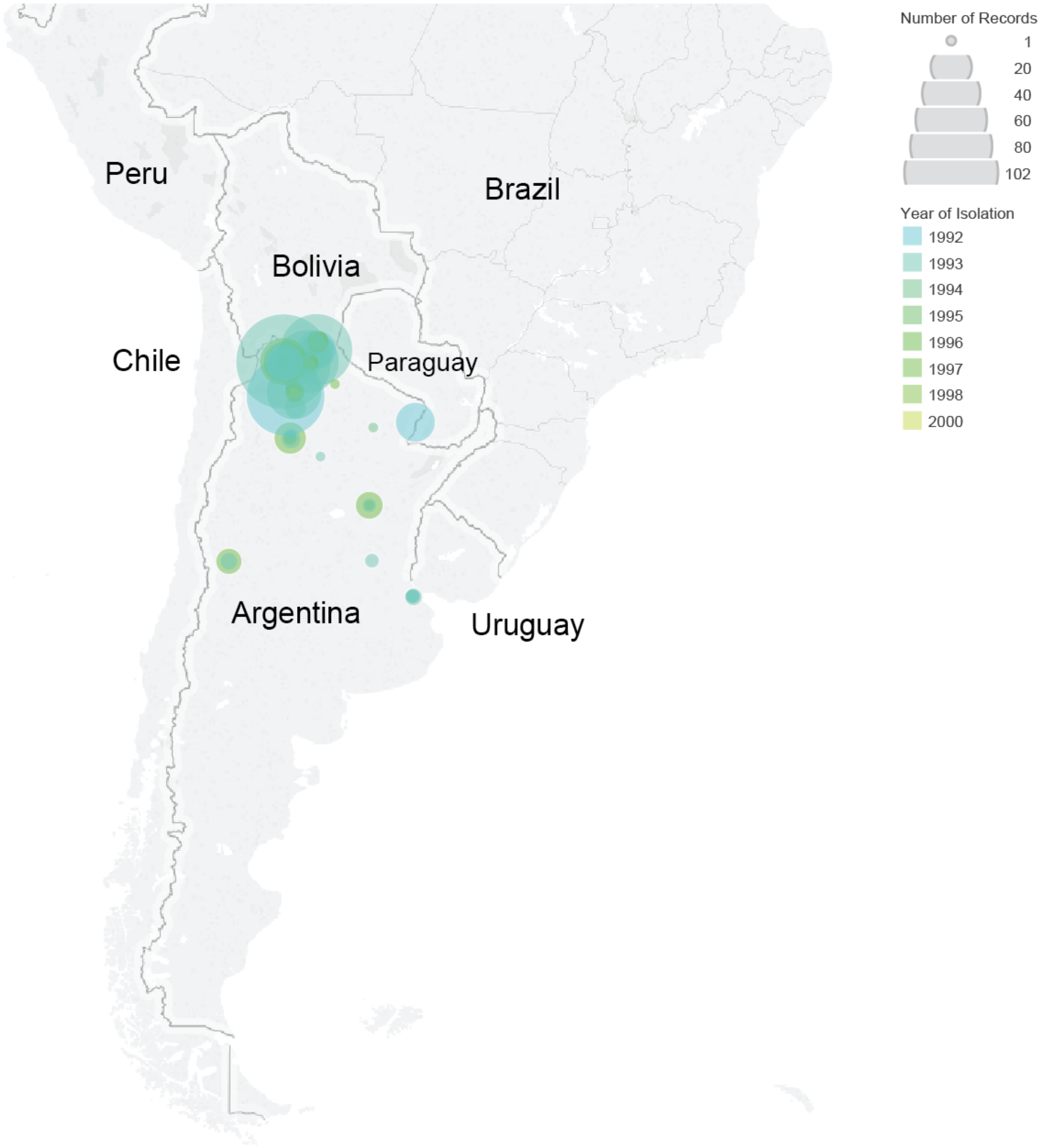

Supplementary Figure 4. An illustration of the recorded geographical origin for isolates sequenced as part of this study (n = 488; Supplementary Data 1-3).

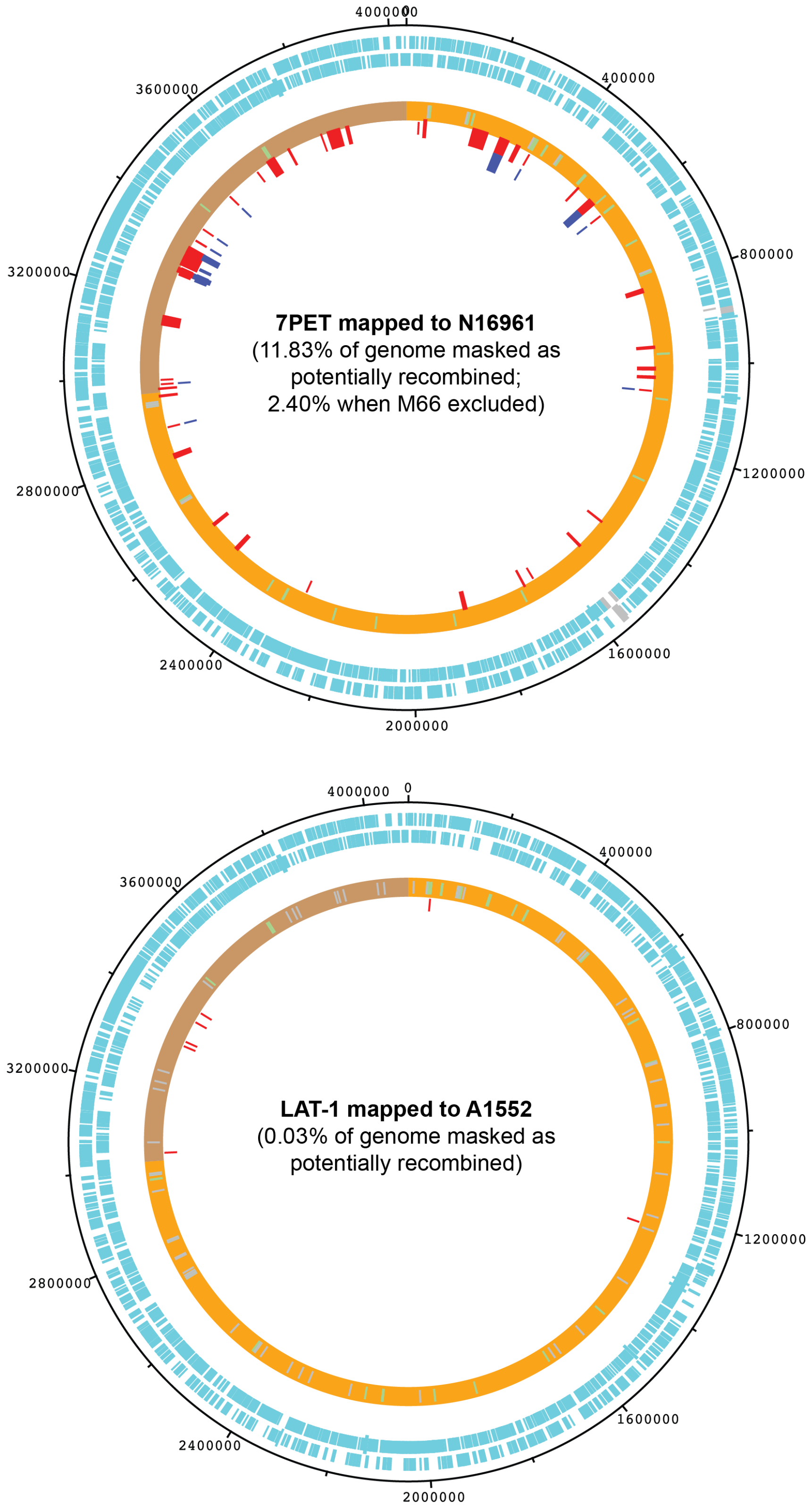

Supplementary Figure 5. Visualisation of the regions of the N16961 and A1552 genome sequences predicted to be recombined. SNVs in putative regions of recombination (red) were

removed from the alignments used to calculate the phylogenetic trees in Figures 2 and 3. The percentage of each genome covered by these putatively recombined regions are reported. The regions in the N16961 genome which were predicted to be recombined, but are not due to variations contributed by the M66 outgroup, are also indicated (blue). The sequences of chromosome 1 and 2 for each genome were concatenated to produce these figures using DNAplotter [9].

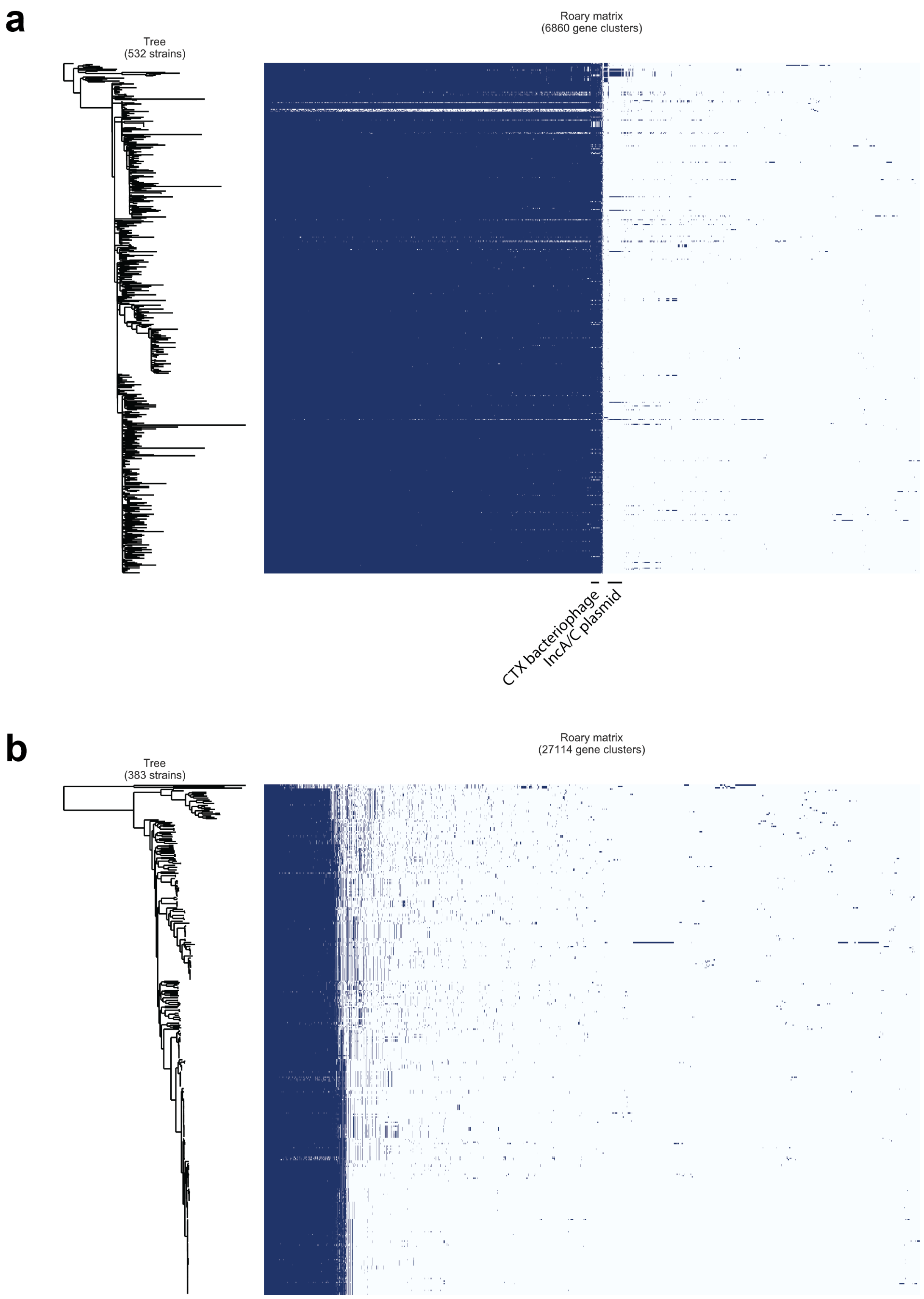

Supplementary Figure 6. Visualisations of the gene presence/absence matrix for LAT-1 genomes (A) and diverse *V. cholerae* (B). The phylogenies presented are the same rooted trees as used in Figure 3A and Figure 4F respectively. The white lines visible in the pangenome matrix in (A) correspond to poorly-assembled genomes.

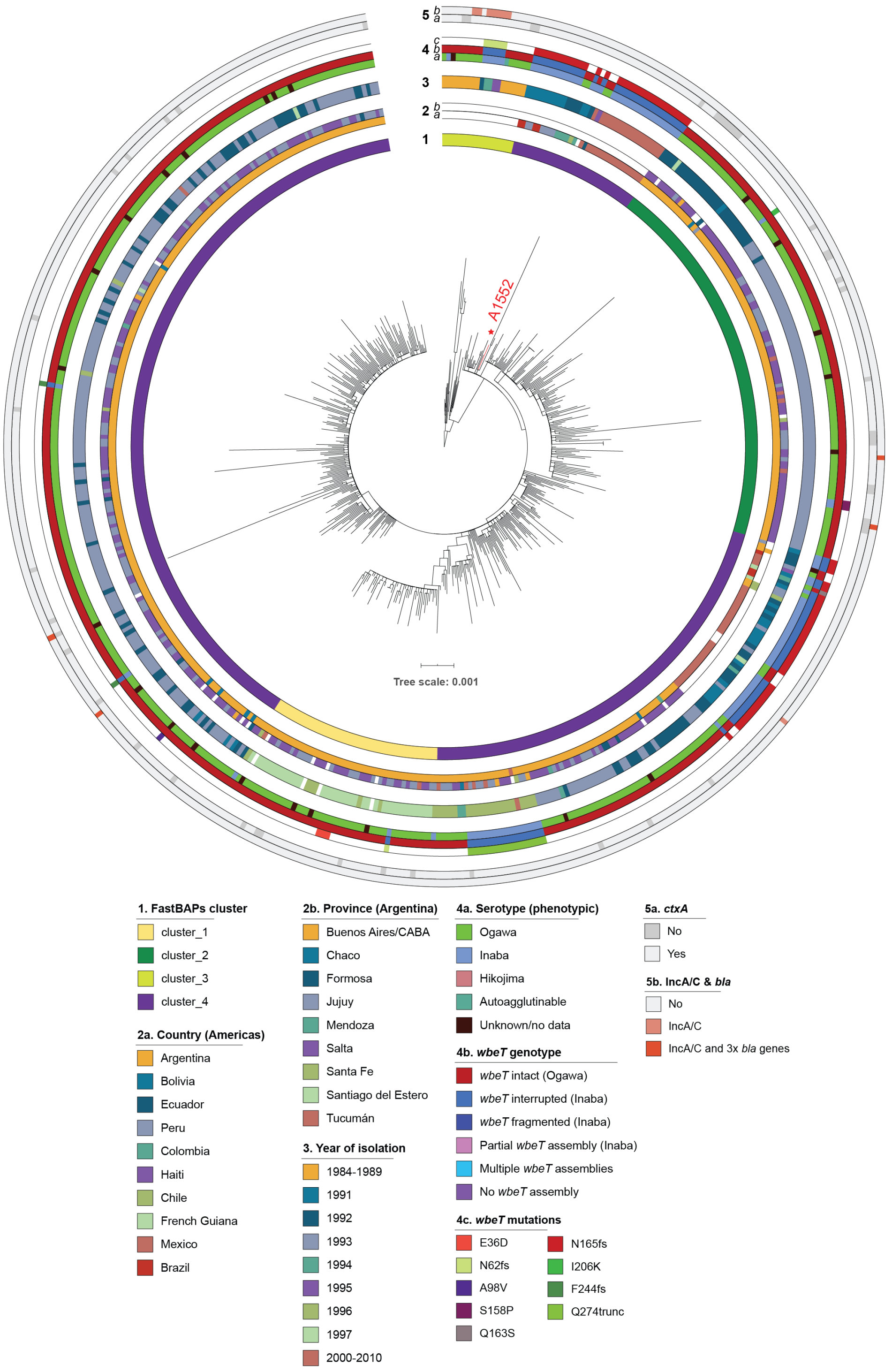

Supplementary Figure 7. Distribution of IncA/C plasmids, and Inaba and Ogawa genotypes and phenotypes within LAT-1. The phylogeny presented is the same as that used in Figure 3A. Mutations in *wbeT*, and the presence of *ctxA*, IncA/C plasmid replicons, and β-lactamase genes were detected *in silico* using ARIBA. The comprehensive results from ARIBA are reported in Supplementary Figure 8. The position of the A1552 reference sequence is indicated (red star).

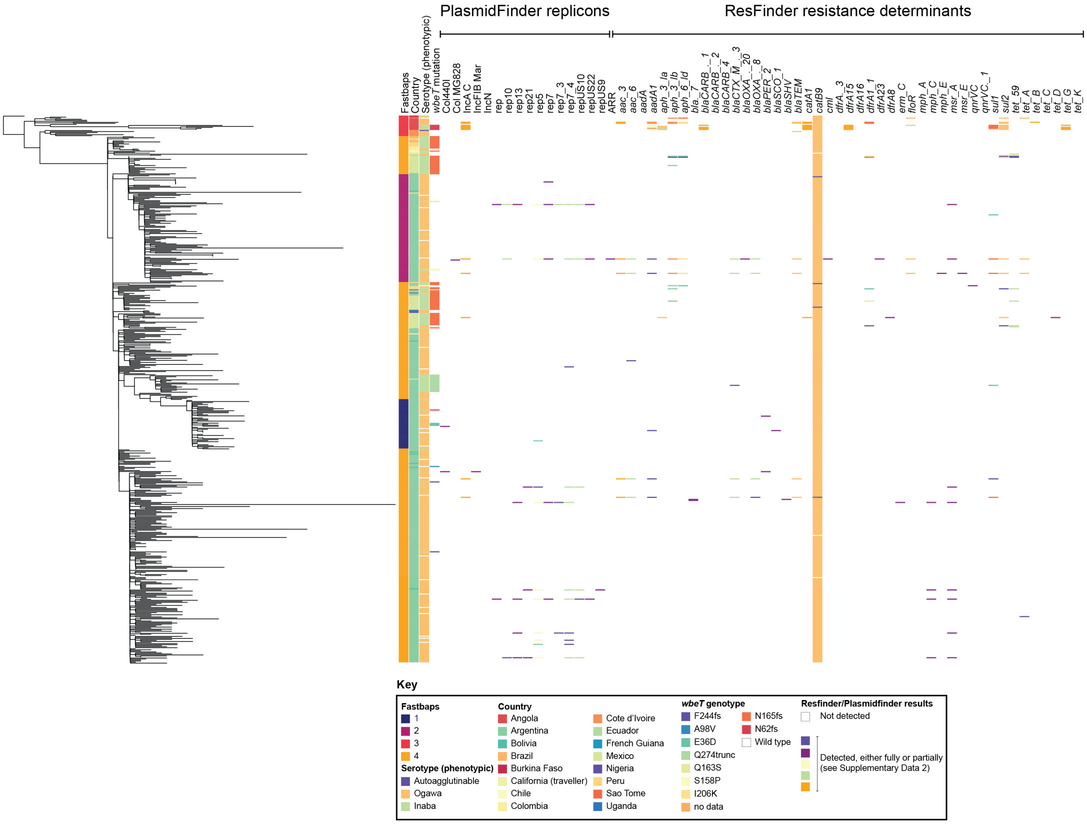

Supplementary Figure 8. ARIBA results visualised using Phandango [10]. The phylogeny presented is the same rooted tree as used in Figure 3A, and the raw data used to produce this figure are available in Supplementary Data 2. All isolates harboured the *catB9* gene, which is known not to be a functional resistance determinant in 7PET *V. cholerae* [11].

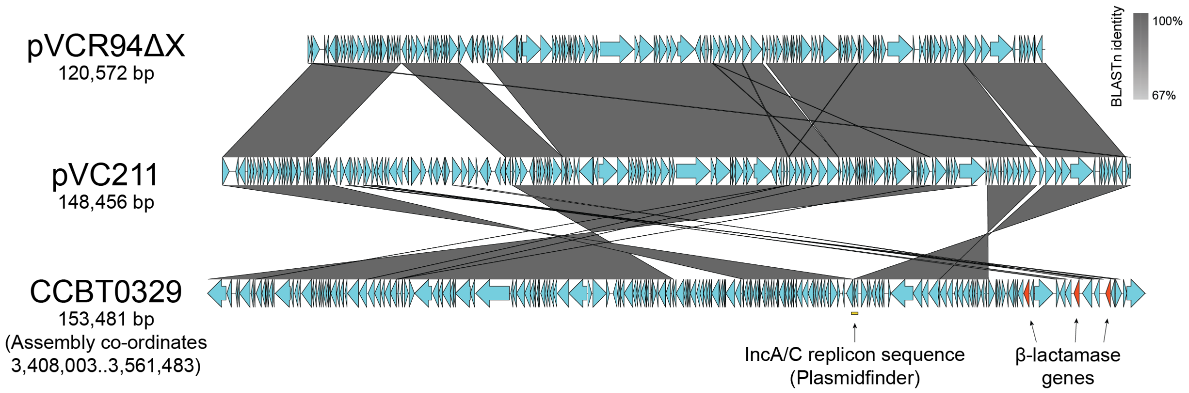

Supplementary Figure 9. An alignment of a single 153,481 contig assembled from the genome sequence of CCBT0329 and the sequences of *V. cholerae* IncA/C plasmids pVC211 [12] and pVCR94ΔX [13] (accessions KY399978.1 and KF551948.1 respectively). Annotations were obtained from Genbank (pVC211, pVCR94ΔX) or from the Prokka annotation (CCBT0329). The genes predicted to encode β-lactamases are indicated (red), as is the region on the CCBT0329 contig containing the IncA/C replicon sequence detected by Plasmidfinder. Figure produced using Easyfig and manually annotated.

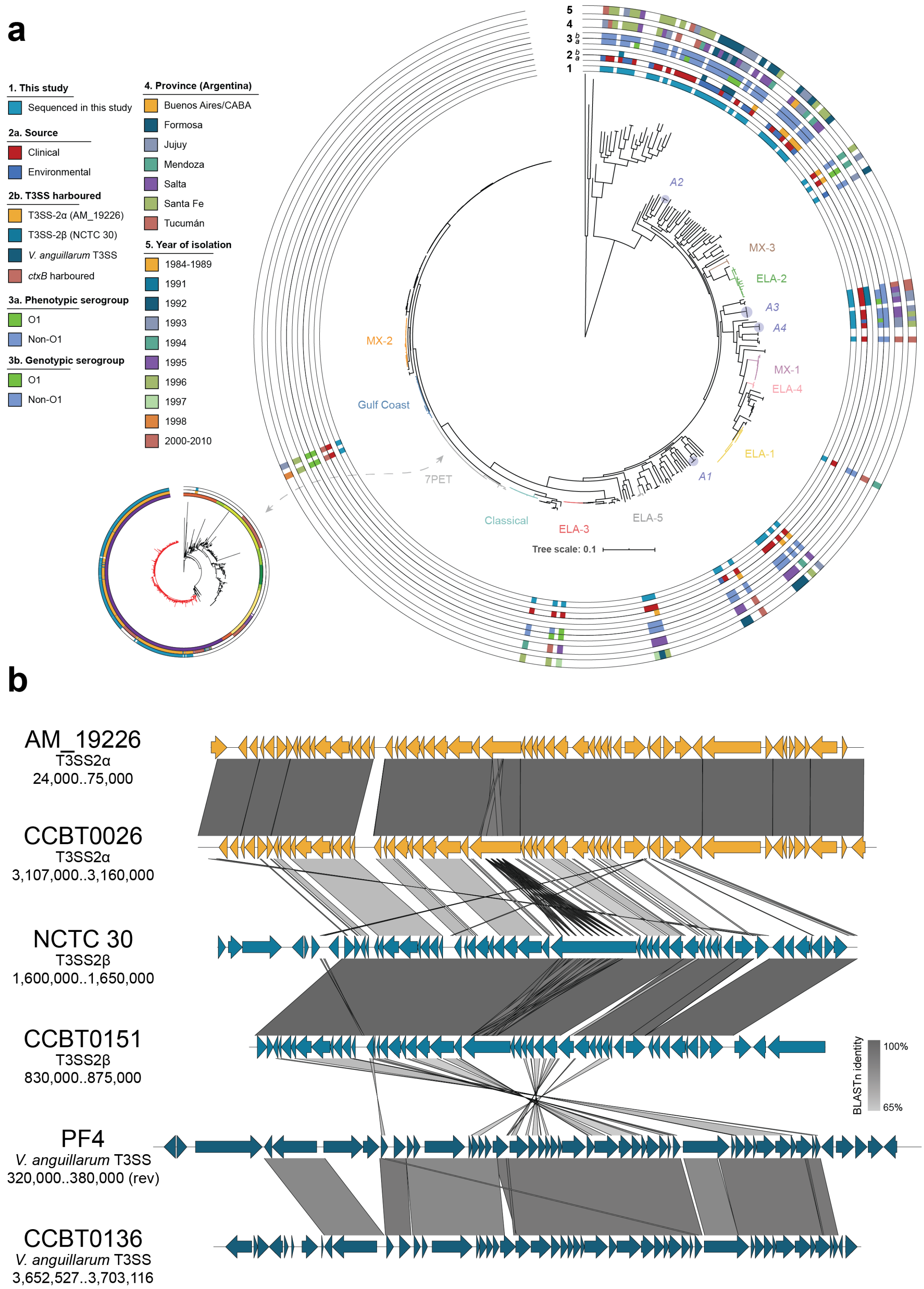

Supplementary Figure 10. Serogroup and T3SS presence across the sequenced non-7PET isolates in this study. Panel (A) depicts the phylogeny presented in Figure 4F, with additional

metadata. Source, province of origin, and year of isolation are presented for the genomes sequenced in this study only. The presence of a T3SS was determined by scanning the pangenome for genes associated with known *V. cholerae* T3SS; three such systems were identified. The presence of *ctxB* was determined both from the pangenome gene presence/absence matrix, and using ARIBA. Serogroup was confirmed *in silico* (see Methods). Exemplar structures of the three T3SS elements identified in these data are presented in (B), and are coloured according to the key presented in (A). The co-ordinates for the subset of each assembly that has been aligned are reported (rev = reverse orientation). Annotations were obtained from Prokka-annotated assemblies used to calculate the pangenome, or from Genbank (PF4 and NCTC 30; accession numbers CP010081.1 and LS997867.1, respectively). The sequence of the T3SS element harboured by NCTC 30 has previously been shown to be that of the T3SS-2β element found in *V. cholerae* 1587 and other sequences that were used in the pangenome analysis [14–16]. Figure produced using Easyfig and manually annotated.

Supplementary References

1. INEI - ANLIS "Dr. Carlos G Malbrán". *WHO global* Salmonella *surveillance América del Sur: Manual de procedimientos: Aislamiento, identificación y caracterización de* Vibrio cholerae. 1–46 http://sgc.anlis.gob.ar/bitstream/123456789/549/1/INEI.manual.vibrio.cholerae.2007.pdf (2007).
2. Rivera, I. N. G. *et al.* Method of DNA extraction and application of multiplex polymerase chain reaction to detect toxigenic *Vibrio cholerae* O1 and O139 from aquatic ecosystems. *Environmental Microbiology* **5**, 599–606 (2003).
3. Chun, J., Huq, A. & Colwell, R. R. Analysis of 16S-23S rRNA intergenic spacer regions of *Vibrio cholerae* and *Vibrio mimicus*. *Applied and Environmental Microbiology* **65**, 2202–2208 (1999).
4. Rivera, I. N. G., Chun, J., Huq, A., Sack, R. B. & Colwell, R. R. Genotypes associated with virulence in environmental isolates of *Vibrio cholerae*. *Applied and Environmental Microbiology* **67**, 2421–2429 (2001).
5. Chow, K. H., Ng, T. K., Yuen, K. Y. & Yam, W. C. Detection of RTX toxin gene in *Vibrio cholerae* by PCR. *Journal of Clinical Microbiology* **39**, 2594–2597 (2001).
6. Carver, T. J. *et al.* ACT: The Artemis comparison tool. *Bioinformatics* **21**, 3422–3423 (2005).
7. Rutherford, K. *et al.* Artemis: Sequence visualization and annotation. *Bioinformatics* **16**, 944–945 (2000).
8. Carver, T., Böhme, U., Otto, T. D., Parkhill, J. & Berriman, M. BamView: Viewing mapped read alignment data in the context of the reference sequence. *Bioinformatics* **26**, 676–677 (2010).

9. Carver, T., Thomson, N., Bleasby, A., Berriman, M. & Parkhill, J. DNAPlotter: Circular and linear interactive genome visualization. *Bioinformatics* **25**, 119–120 (2009).

10. Hadfield, J. *et al.* Phandango: An interactive viewer for bacterial population genomics. *Bioinformatics* **34**, 292–293 (2018).

11. Weill, F.-X. *et al.* Genomic history of the seventh pandemic of cholera in Africa. *Science* **358**, 785–789 (2017).

12. Wang, R., Liu, H., Zhao, X., Li, J. & Wan, K. IncA/C plasmids conferring high azithromycin resistance in *Vibrio cholerae*. *International Journal of Antimicrobial Agents* **51**, 140–144 (2018).

13. Carraro, N. *et al.* Development of pVCR94ΔX from *Vibrio cholerae*, a prototype for studying multidrug resistant IncA/C conjugative plasmids. *Frontiers in Microbiology* **5**, (2014).

14. Dorman, M. J. *et al.* The history, genome and biology of NCTC 30: a non-pandemic *Vibrio cholerae* isolate from World War One. *Proceedings of the Royal Society B: Biological Sciences* **286**, 20182025 (2019).

15. Carpenter, M. R. *et al.* CRISPR-Cas and contact-dependent secretion systems present on excisable pathogenicity islands with conserved recombination modules. *Journal of Bacteriology* **199**, e00842-16 (2017).

16. Miller, K. A., Tomberlin, K. F. & Dziejman, M. *Vibrio* variations on a type three theme. *Current Opinion in Microbiology* **47**, 66–73 (2019).
