# Supplementary material for: Genomics of the Argentinian cholera epidemic elucidate the contrasting dynamics of epidemic and endemic Vibrio cholerae
Source: Nat Commun. 2020 Oct 1;11:4918. doi: 10.1038/s41467-020-18647-7 (PMC7530988; doi:10.1038/s41467-020-18647-7)
Supplement: Supplementary file 5 — Reporting summary [file 41467_2020_18647_MOESM5_ESM.pdf]

nature research

Corresponding author(s): Josefina Campos and Nicholas R Thomson

Last updated by author(s): Aug 14, 2020

Reporting Summary

Nature Research wishes to improve the reproducibility of the work that we publish. This form provides structure for consistency and transparency in reporting. For further information on Nature Research policies, see Authors & Referees and the Editorial Policy Checklist .

Statistics

For all statistical analyses, confirm that the following items are present in the figure legend, table legend, main text, or Methods section.

n/a	Confirmed	
☐	☒	The exact sample size (*n*) for each experimental group/condition, given as a discrete number and unit of measurement
☒	☐	A statement on whether measurements were taken from distinct samples or whether the same sample was measured repeatedly
☒	☐	The statistical test(s) used AND whether they are one- or two-sided<br>*Only common tests should be described solely by name; describe more complex techniques in the Methods section.*
☒	☐	A description of all covariates tested
☒	☐	A description of any assumptions or corrections, such as tests of normality and adjustment for multiple comparisons
☐	☒	A full description of the statistical parameters including central tendency (e.g. means) or other basic estimates (e.g. regression coefficient) AND variation (e.g. standard deviation) or associated estimates of uncertainty (e.g. confidence intervals)
☒	☐	For null hypothesis testing, the test statistic (e.g. *F*, *t*, *r*) with confidence intervals, effect sizes, degrees of freedom and *P* value noted<br>*Give P values as exact values whenever suitable.*
☐	☒	For Bayesian analysis, information on the choice of priors and Markov chain Monte Carlo settings
☒	☐	For hierarchical and complex designs, identification of the appropriate level for tests and full reporting of outcomes
☒	☐	Estimates of effect sizes (e.g. Cohen's *d*, Pearson's *r*), indicating how they were calculated

Our web collection on statistics for biologists contains articles on many of the points above.

Software and code

Policy information about availability of computer code

Data collection	No software was used for data collection
Data analysis	A complete description of the data analyses performed in this study is reported in Methods. No custom programs were used in this study.<br><br>The following software was used in this study:<br>assembly-stats v1.0.1<br>Kraken v0.10.6<br>SPAdes v3.8.2<br>Prokka v1.5<br>Roary v3.12.0<br>SMALT v0.7.4<br>Gubbins v1.4.10<br>SNP-sites v2.4.1 and v2.5.1<br>trimAl v1.4.1<br>IQ-Tree v1.6.10<br>Fastbaps v1.0.1<br>extract_PI_SNPs.py (no version number; https://gist.github.com/jasonsahl/9306cd014b63cae12154)<br>snp-dists v0.4<br>FastANI v1.0<br>ARIBA v2.12.1<br>Tableau Desktop 2018.31<br>Figtree v1.4.3<br>iTOL v3

roary_plots.py v0.1.0
R v3.5.1
ggplot2 v3.1.1
reshape v0.8.8
Artemis v16
ACT v13
DNAplotter v1.11
Easyfig v2.2.2
iCANDY (no version number; https://github.com/simonrharris/iCANDY)
Adobe Illustrator CC v23.0.4
BAMview v1.2 (part of Artemis v16)
OpenStreetMap (accessed in 2019 and 2020 via Tableau Desktop 2018.31)

Code availability:

The R code to produce Figures 3B and 4E is available in Figshare, https://dx.doi.org/10.6084/m9.figshare.11310131. No custom programs were used in this study

For manuscripts utilizing custom algorithms or software that are central to the research but not yet described in published literature, software must be made available to editors/reviewers. We strongly encourage code deposition in a community repository (e.g. GitHub). See the Nature Research guidelines for submitting code & software for further information.

Data

Policy information about availability of data

All manuscripts must include a data availability statement. This statement should provide the following information, where applicable:

- Accession codes, unique identifiers, or web links for publicly available datasets
- A list of figures that have associated raw data
- A description of any restrictions on data availability

A data availability statement is included in the manuscript.

All next-generation sequencing data generated in this study have been deposited into the European Nucleotide Archive (ENA; http://www.ebi.ac.uk/ena) under accession number ERP118963 [https://www.ebi.ac.uk/ena/browser/view/PRJEB35844].

The assemblies which were used to produce Supplementary Figures 9 and 10B have been deposited into the ENA as part of ERP118963 [https://www.ebi.ac.uk/ena/browser/view/PRJEB35844].

The following publicly-available reference genome sequences were used in this study:

The N16961 V. cholerae isolate sequence (LT907989 [https://www.ebi.ac.uk/ena/browser/view/LT907989]/LT907990 [https://www.ebi.ac.uk/ena/browser/view/LT907990])
The A1552 V. cholerae isolate sequence CP025936 [https://www.ebi.ac.uk/ena/browser/view/CP025936]/CP025937 [https://www.ebi.ac.uk/ena/browser/view/CP025937])
The NCTC 9420 V. cholerae isolate sequence (CP013319 [https://www.ebi.ac.uk/ena/browser/view/CP013319]/CP013320 [https://www.ebi.ac.uk/ena/browser/view/CP013320]),
The Classical V. cholerae O395 isolate sequence (CP000626 [https://www.ebi.ac.uk/ena/browser/view/CP000626]/CP000627 [https://www.ebi.ac.uk/ena/browser/view/CP000627]).
The WbeT protein sequence from the Ogawa V. cholerae isolate VX44945 (AEN80191.1 [https://www.ebi.ac.uk/ena/browser/view/AEN80191.1]).

Complete lists of accession numbers for sequences produced in this study as well as previously-published genome sequences used for phylogenetic analysis (total number = 1,318, non-redundant) are provided in Supplementary Data 1-3.

The original data which underpin Figure 1 are held by INEI and are available on request (J.C.).

All other data used to generate figures in this manuscript, including sequence alignments, phylogenetic trees, and data matrices, are available in Figshare (https://dx.doi.org/10.6084/m9.figshare.11310131) or in the Supplementary Data associated with this paper.

An interactive LAT-1 phylogeny is available in Microreact (https://microreact.org/project/VAZD_K0kZ).

Field-specific reporting

Please select the one below that is the best fit for your research. If you are not sure, read the appropriate sections before making your selection.

☐ Life sciences ☐ Behavioural & social sciences ☒ Ecological, evolutionary & environmental sciences

For a reference copy of the document with all sections, see nature.com/documents/nr-reporting-summary-flat.pdf

Ecological, evolutionary & environmental sciences study design

All studies must disclose on these points even when the disclosure is negative.

Study description	This study used archived bacterial cultures from the collection of Vibrio cholerae held at INEI-ANLIS "Dr. Carlos G. Malbrán", the Argentinian national reference laboratory for enterobacteria. In this study, we sequenced the genomes of as many V. cholerae as was practical, producing a total of 490 V. cholerae genome sequences, together with associated metadata. The study was designed to study the evolution of a bacterial pathogen using isolates that were collected as a by-product of national cholera surveillance in Argentina. Please note that this study used the genomes of archived bacteria and summary statistics related to the receipt of these bacteria at INEI. At no point were any patient data or other identifiable data used in, or made available for, this study. This has been stated explicitly in the 'Ethics' section of the manuscript.
Research sample	We used a set of 490 Vibrio cholerae from the INEI-ANLIS bacterial culture archive, together with phenotypic metadata and region/ date of origin pertaining to each isolate. These data were contextualised with previously-published, publicly-available genome sequences for other V. cholerae.<br><br>These samples were chosen principally to capture diversity of both O1 and non-O1 V. cholerae at the beginning (1992-1993) and the end (1996-1997) of the Argentinian cholera epidemic (1992-1998). The sequenced isolates were a spatiotemporally-broad cross-section of cholera incidence - they were obtained from all regions of Argentina that experienced cholera cases. They were also chosen to capture apparent shifts between Inaba and Ogawa serotype.<br><br>The datasets used in this study were either generated by whole-genome sequencing of bacterial isolates, or comprised previously-published genome sequences that are available in publicly-accessible databases. Accession numbers for all publicly-available sequence data are listed in the manuscript, in this Reporting Summary, and in the Supplementary Data.
Sampling strategy	No sample size calculations were performed. We sequenced as many viable bacterial isolates as was practical. Limitations were imposed by available resources, and by the fact that not all of the archived cultures were viable.
Data collection	The metadata associated with each bacterial isolate were recorded by INEI staff at the time of receiving the isolate at the reference laboratory. These included the results of serotyping, PCR, biochemical, and other phenotypic tests as listed in the manuscript (Methods) and Supplementary Data 1-3. The dates and places of isolation were recorded by the laboratories submitting cultures to INEI for verification. The collection of these metadata occurred between 1992 and the early 2000s, at and around the time of the isolate's receipt at the reference laboratory. These metadata were aggregated for this study.<br><br>The new genome sequences used in the study were generated by the core sequencing teams at the Wellcome Sanger Institute. Previously-published genome sequences were aggregated from the publications listed in Supplementary Data 1-3: these data were downloaded from the European Nucleotide Archive using the accession numbers listed in Supplementary Data 1-3.
Timing and spatial scale	Isolates were chosen from the INEI-ANLIS collection to cover the beginning and end of the 1990s cholera epidemic in Argentina. V. cholerae recorded as being both serogroup O1 and non-O1 were included, with the aim of studying the background of non-epidemic (non-O1) V. cholerae that co-existed alongside the epidemic (O1) lineage in the country.<br><br>The earliest isolate was obtained on 10th February 1992 and the latest isolate was obtained on 18th August 2005. Six isolates were obtained outside of the epidemic period, the earliest of which was obtained on 3rd February 2000 and the latest of which on 18th August 2005 (see Supplementary Data 3). The latest isolate from within the epidemic period was obtained on 23rd February 1998. Isolates were sent to INEI-ANLIS from suspected cases of cholera, accordingly, there was no periodicity or specific frequency of sampling other than that of the natural progression of cholera epidemics in Argentina (see Figure 1). Isolates were included from all geographic regions that suffered from cholera during the epidemic period. This was concentrated to the North of Argentina (see Figure 1) but spanned an area of approximately 1.2 million square kilometres.
Data exclusions	The exclusion of data is explicitly described in the 'Methods' section of the manuscript: "A total of 21 sequenced isolates contained substantial amounts of contaminating sequences from non-Vibrio species, and were excluded from this study, for a total of 490 sequences used in this analysis. Contamination was assessed using Kraken v0.10.6, by examining the overall length of the SPAdes assembly (data were summarised using assembly-stats v1.0.1 (https://github.com/sanger-pathogens/assembly-stats) and assemblies greater than 5 Mbp in length were excluded) and by inspection of initial phylogenetic trees.". The contaminated genome sequences have not been published alongside this manuscript.
Reproducibility	Replication of sequencing was not performed; every bacterial isolate was sequenced once. However, by using next-generation sequencing technology, sequencing an isolate once actually means that each genome is sequenced many times. The target sequencing depth per isolate was 30 X - i.e., the sequencing data obtained for each isolate should cover the genome of that isolate at least 30 times. Thus, within a sequencing run, each isolate is sequenced the equivalent of 30 times. Consensus sequences for each isolate were used for all genome assemblies and SNV identification (see Methods). Therefore, although DNA from each isolate was only submitted once for sequencing, the genome of the isolate was sequenced multiple times.
Randomization	This study was not randomised. Sequences were allocated into '7PET', 'LAT-1', and 'non-7PET' groups on the basis of their phylogenetic position.

Blinding	Initial phylogenetic analyses were carried out without being influenced by metadata - place/date of origin, serogroup, etc., that is to say, construction of these preliminary phylogenetic trees was agnostic of the group to which each sample belonged, and was blind to the metadata associated with each sample. Once phylogenies had been constructed and sequences had been classified, all metadata were then included in analyses where appropriate.

Did the study involve field work? ☐ Yes ☒ No

Reporting for specific materials, systems and methods

We require information from authors about some types of materials, experimental systems and methods used in many studies. Here, indicate whether each material, system or method listed is relevant to your study. If you are not sure if a list item applies to your research, read the appropriate section before selecting a response.

Materials & experimental systems

n/a	Involved in the study	
☒	☐	Antibodies
☒	☐	Eukaryotic cell lines
☒	☐	Palaeontology
☒	☐	Animals and other organisms
☒	☐	Human research participants
☒	☐	Clinical data

Methods

n/a	Involved in the study	
☒	☐	ChIP-seq
☒	☐	Flow cytometry
☒	☐	MRI-based neuroimaging
